# Towards *operando* computational modeling in heterogeneous catalysis

**DOI:** 10.1039/c8cs00398j

**Published:** 2018-09-11

**Authors:** Lukáš Grajciar, Christopher J. Heard, Anton A. Bondarenko, Mikhail V. Polynski, Jittima Meeprasert, Evgeny A. Pidko, Petr Nachtigall

**Affiliations:** a Department of Physical and Macromolecular Chemistry , Faculty of Science , Charles University in Prague , 128 43 Prague 2 , Czech Republic . Email: lukas.grajciar@natur.cuni.cz ; Email: petr.nachtigall@natur.cuni.cz ; Email: heardc@natur.cuni.cz; b TheoMAT group , ITMO University , Lomonosova 9 , St. Petersburg , 191002 , Russia; c Inorganic Systems Engineering group , Department of Chemical Engineering , Faculty of Applied Sciences , Delft University of Technology , Van der Maasweg 9 , 2629 HZ Delft , The Netherlands . Email: e.a.pidko@tudelft.nl

## Abstract

An increased synergy between experimental and theoretical investigations in heterogeneous catalysis has become apparent during the last decade. Experimental work has extended from ultra-high vacuum and low temperature towards *operando* conditions. These developments have motivated the computational community to move from standard descriptive computational models, based on inspection of the potential energy surface at 0 K and low reactant concentrations (0 K/UHV model), to more realistic conditions. The transition from 0 K/UHV to *operando* models has been backed by significant developments in computer hardware and software over the past few decades. New methodological developments, designed to overcome part of the gap between 0 K/UHV and *operando* conditions, include (i) global optimization techniques, (ii) *ab initio* constrained thermodynamics, (iii) biased molecular dynamics, (iv) microkinetic models of reaction networks and (v) machine learning approaches. The importance of the transition is highlighted by discussing how the molecular level picture of catalytic sites and the associated reaction mechanisms changes when the chemical environment, pressure and temperature effects are correctly accounted for in molecular simulations. It is the purpose of this review to discuss each method on an equal footing, and to draw connections between methods, particularly where they may be applied in combination.

## Abstract 2

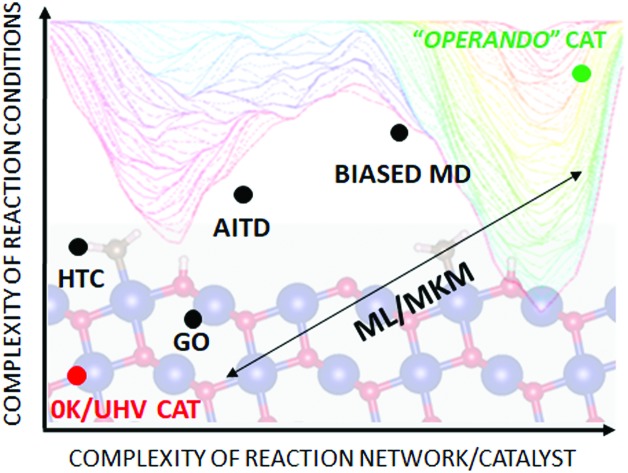
An increased synergy between experimental and theoretical investigations in heterogeneous catalysis has become apparent during the last decade.

## 1. Introduction

Most of the chemicals produced nowadays are obtained using processes based on catalysis. The on-going search for optimal process conditions and the most suitable catalyst is driven by various concerns, including (i) environmental impact, (ii) resource utilization, (iii) safety and (iv) overall process economy. While this has traditionally been the domain of experimental investigations, the input from computational investigations has been steadily increasing over the last 40 years. An increased synergy between theory and experiment has become apparent during the last decade, in particular, in the field of heterogeneous catalysis.

By definition a heterogeneous catalyst shifts the reference reaction onto a different free energy surface where the energy of critical transition states with respect to relevant intermediates becomes lower. Mechanisms of chemical reactions were traditionally explored within the concept of the potential energy surface (PES), considering simplified models of a catalytic system working under idealized conditions of, basically, infinite dilution. Such a heterogeneous catalysis model represents ultra-high vacuum conditions, for which calculations provide information at 0 K; we will refer to this model as the 0 K/UHV model. Strictly speaking, such a description corresponds to rather unrealistic reaction conditions and its validity decreases with increasing temperature and pressure. A great number of mechanisms have been proposed based on calculations with such a simplistic model and results were often at least in qualitative agreement with available experimental data. Computational results obtained with 0 K/UHV model correspond reasonably well with experimental data obtained for well-defined surfaces under UHV conditions. However, the overlap of such calculated data and catalytic experiments carried out under realistic conditions is rather small, and a good agreement between 0 K/UHV theory and catalytic experiments was often just fortuitous.

The success of the simple PES concept applied within the 0 K/UHV approximation can be expected only when the following assumptions hold: (i) the structure of the active site under realistic conditions is known (or correctly guessed), (ii) both the structure of the active site and the reaction mechanism do not depend on the surface coverage of individual reaction intermediates, (iii) the reaction mechanism found under nearly UHV conditions is not different from that at the realistic composition of the surrounding gas or liquid phase and (iv) temperature effects, including the transition from PES to free energy surface (FES), can be safely neglected. Unfortunately, all such assumptions are rarely satisfied at once. If the temperature is relatively low it follows that reactants, products and/or reaction intermediates are adsorbed on the surface; and in contrast, one can expect that the reaction proceeds on a clean catalyst surface only at elevated temperature.

A deeper atomistic insight into the reaction mechanisms, the catalyst structure/activity relationship and catalyst stability/transformation during the reaction greatly increases our chances to find the optimal catalyst for a particular process. The most detailed experimental evidence about the catalyst at the molecular level can be obtained by a combination of characterization techniques under UHV conditions. More and more information becomes available from experimental investigations gathered under the conditions of a model catalytic reaction – *in situ* conditions – and also under conditions where the applied catalytic process takes place – *operando* conditions. For details of experimental *in situ* and *operando* conditions see, *e.g.*, ref. 1–3. A great development of *in situ* and in particular *operando* experimental techniques for studying catalytic reactions in the last 20 years has brought an increasing amount of information about the state of the catalysts under realistic conditions.^4^,^5^

Among the most important findings emerging from such studies is the evidence of the dynamic nature of the catalyst surface, whose structure constantly changes under the catalytic reaction conditions. For example, in oxidation catalysis by supported metal nanoparticles, *in situ* and *operando* techniques revealed the formation of ultra-thin oxide layers covering the metal nanoparticles in an oxidizing atmosphere, which provide the active sites for the target catalytic reactions. Obviously, such an active site model could not be proposed based on the UHV surface science experiments or computations carried out in the 0 K/UHV regime. A problem of how the structure of the catalyst depends on the realistic chemical environment and temperature that are relevant for a particular process is thus the key for a proper understanding of catalysis at the molecular level and for a design of improved catalysts.^6^–^8^

**Fig. 1:**
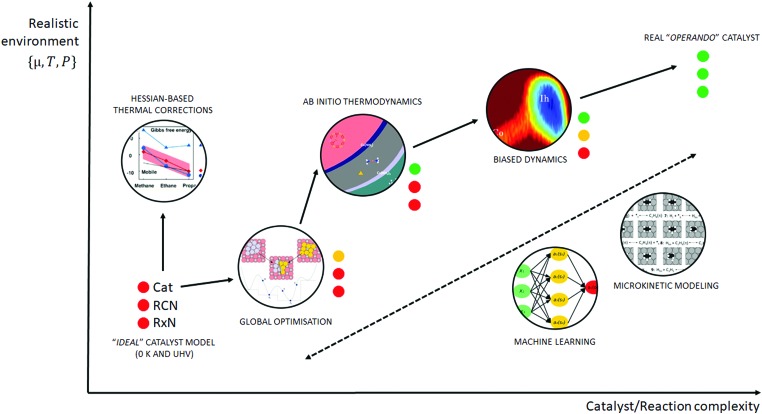
Schematic of the various computational methods applied to heterogeneous catalysis, which lie between an idealised UHV model and a realistic, *operando* model. The traffic light key depicts the quality of each method with respect to catalyst model complexity (Cat), reaction coordinate accuracy (RCN) and reaction network complexity (RxN). Adapted with permission from Piccini *et al.*, *Journal of Physical Chemistry C*, 2015, **119**, 6128–6137, Copyright 2015, American Chemical Society, Vilhelmsen *et al.*, *Journal of Chemical Physics*, 2014, **141**, 044711, Copyright 2014, American Institute of Physics, Chen *et al.*, *Journal of Catalysis*, 2018, **358**, 179–186, Copyright 2018, Elsevier, Pavan *et al.*, *Journal of Chemical Physics*, 2015, **143**, 184304, Copyright 2015, American Institute of Physics, Heard *et al.*, *ACS Catalysis*, 2016, **6**, 3277–3286, Copyright 2016, American Chemical Society.

Similar to the shift of experimental investigations in catalysis from UHV to *operando* conditions, theoretical investigations in the field of catalysis are moving more and more from 0 K/UHV models to computational *operando* investigations. In analogy with the experimental *operando* conditions, a computational *operando* model is defined by the following conditions: the structure of the active catalyst surface and the reaction coordinates must reflect realistic conditions during the reaction and a complex reaction network must be established (see Fig. 1 and corresponding text for more details). However, a transition from the 0 K/UHV to *operando* model dramatically influences the complexity of the problem and increases computational demands. A number of methods have been developed in the past few decades that ease the 0 K/UHV → *operando* transition and it is the goal of this review to discuss the current state of the computational investigations of catalysis, with the goal to enable the long-sought after paradigm of catalysis by design.

A huge gap between the 0 K/UHV models on one side and *operando* models on the other side cannot be overcome by a single computational method that would explicitly account for the whole complexity of the underlying phenomena. A multiscale modeling approach can be followed to construct a composite methodology that includes all the crucial physical phenomena. In our opinion, the following five methods appear to be the most important for bridging this gap: (i) global optimization techniques, (ii) *ab initio* constrained thermodynamics, (iii) biased MD simulations, (iv) microkinetic models of reaction networks. The fifth class of methods is a conceptually different approach that does not necessarily imply the explicit account of the complex physics of a catalyst system and yet holds great promise as a tool to enable catalysis by design. This class is the broad family of machine learning methods. The latest development of each of these five techniques is addressed individually in the following five sections of this review.

A transition from the 0 K/UHV to *operando* model is schematically depicted in Fig. 1. The 0 K/UHV model corresponds to the situation at the lower left corner, corresponding to vanishing partial pressures of reaction components (expressed in terms of chemical potentials) and low temperature. The *operando* model corresponds to the upper right corner. Going from bottom to top of the figure the reaction environment (in terms of chemical potentials and temperature) becomes more realistic. Any model improvement results in the increased complexity of the problem (from left to right), mostly in the number of configurations that are considered. Basics of the 0 K/UHV model include the following approximations: (i) idealized catalyst surface (denoted as Cat in Fig. 1), (ii) idealized reaction coordinates with minimum number of reactants on the PES at 0 K (reaction coordinate environment – RCE) and (iii) elementary reaction steps are considered (reaction network – RxN). All these approximations must be lifted to move forward to an *operando* model.

Methods presented in Fig. 1 from left to right start with Hessian-based thermal corrections, followed by a global optimization approach, *ab initio* constrained thermodynamics and biased MD; microkinetic modeling and machine learning techniques are taken off this order since they can be used at any level of the 0 K/UHV → *operando* transition. The order presented in Fig. 1 is motivated by the fact that if all extensions are applied for a particular system, they would be applied in the order presented in the figure, with the exception of Hessian-based thermal corrections. Hessian-based thermal corrections allow a proper transition from potential- to free-energy surfaces while the complexity of the system remains unchanged; they can be used either to improve the 0 K/UHV model or in combination with global optimization or *ab initio* constrained dynamics (improving the quality of partition functions). It is important to note that it is common to apply just one or two extensions (or even three in some cases) and by no means does it have to be the first methods from left to right. For example, it is rather common to combine Hessian-based thermal corrections directly with microkinetics. It depends on the particular problem under investigation as to which of the extensions is crucial. Global optimization techniques mostly help in finding relevant configurations when these are difficult or impossible to obtain from relevant experimental data. *Ab initio* thermodynamics is critically important for the investigation of catalyst surfaces that are changed in the reaction environment. Biased molecular dynamics (MD) techniques become essential for the localization of transition states in complex environments when these are strongly affected by the surrounding molecules. Microkinetic modeling of the reaction network is essential for situations in which a large number of reaction intermediates exist. Last but not least, machine learning techniques are emerging as a useful tool in rationalization of the system descriptors and finding important correlations in large data sets.

Each of the methods presented in Fig. 1 is designed to overcome part of the gap between 0 K/UHV and *operando* conditions. Each method is discussed in the following sections and each of the methods has been reviewed separately in recent years in a comprehensive way. It is the purpose of this review to discuss them on an equal footing with respect to the gap between 0 K/UHV and *operando*. It should be stressed that the simultaneous application of all these extensions is computationally prohibitive in a general sense. But it should be noted that it is often not necessary to apply all these model extensions for a particular catalytic system; instead it is important to identify which of the extensions is critical for the problem investigated.

## 2. Global optimization

### 2.1. Basic principles

Global optimization (GO) is a class of heuristic methods used to search the breadth of a cost function *E*(***X***) defined by the multidimensional vector ***X***. The goal is to locate the local minimum ***X***[combining tilde], which globally minimizes that function, such that *E*(***X***[combining tilde]) = min [*E*(***X***)]. In the case of computational chemistry, ***X*** is usually a 3N dimensional vector of the atomic coordinates of an N atom system, which returns the potential energy of the configuration. Thus, GO is aimed at finding the structure which has the lowest potential energy. The dimensionality of the landscape to be sought is often reduced considerably from 3N, either by constraining the position of certain atoms during local minimisation, by combining positions into collective variables (as in metadynamics, described in detail in Section 4), or by defining symmetry classes to group like atoms together. As it is impossible in practice to ensure that a putative global minimum is the true global minimum for all but trivial systems, convergence criteria on GO searches are applied, based either on stagnation of the diversity of structures, or some practical consideration, such as the number of minima found. The moveclass, by which the search of the energy landscape is undertaken, is another heuristic choice, described for a number of GO methods in Section 2.2. Combining a moveclass, a local minimisation algorithm, an acceptance criterion for new structures, and a convergence criterion for the search, a GO method can locate a library of low-lying minima relevant for the physico-chemical property of interest, or deliver the global minimum, to be used as the best estimate of the preferred structure of a system.

### 2.2. Global optimization methods

In condensed matter physics, we are primarily concerned with finding the stable phases of materials, and as such GO has been widely applied. GO provides a library of low energy configurations, which are useful for describing systems with strong structure–function relationships. In heterogeneous catalysis, the picture is more complicated, as catalytic reactions are often controlled kinetically, rather than thermodynamically, and involve transient species and dynamic restructuring of the active phase. Generally, low temperature, low pressure environments are most accurately reproduced by GO techniques. Hence, from the first reports of robust methods in the 1990s, GO has been developed and applied successfully in heterogeneous catalysis research in three main areas: (i) the optimization of vacuum phase nanoparticle and cluster structures, (ii) determination of stable, catalytically important surfaces and (iii) the adsorption, growth and migration of active catalytic particles upon substrates, which usually aim to connect to gas phase spectroscopy or surface science experiments. The variety of GO techniques will be covered in the following section, supported by appropriate examples that are relevant for catalysis. For systems which change strongly in structure or composition during a reaction, or interact strongly with the environment, GO is less valuable, which is why it is seldom applied, for example, to *operando* descriptions of systems with complex solvation chemistry.

A good GO method must balance the local and global aspects of searching the energy landscape. Local optimization methods serve to locate the configuration which corresponds to the local minimum of the potential energy well to which the current configuration belongs. The computational methods to achieve this are numerous and robust.^9^,^10^ However, the global search is required to explore the breadth of the energy landscape efficiently, so as to capture all relevant structural classes. This is performed in a heuristic, system-specific manner. In a recent article by Jørgensen and colleagues, the balance between efficient global and local search is recast into the concept of “exploration *versus* exploitation”.^11^ They find that the optimal balance between exploration (finding new regions of configuration space) and exploitation (exhausting the local region to find all nearby low-lying minima) can enhance the GO efficiency for molecular structures. By contrast, it is found to be less powerful for surface GO, because the possible configurations are strongly templated by the layers below. Several good reviews exist for detailed examination of the technical aspects of global landscape search and optimization methods.^12^,^13^ We will give a brief introduction to the more popular techniques, before describing the catalytic applications in more detail.

**Fig. 2:**
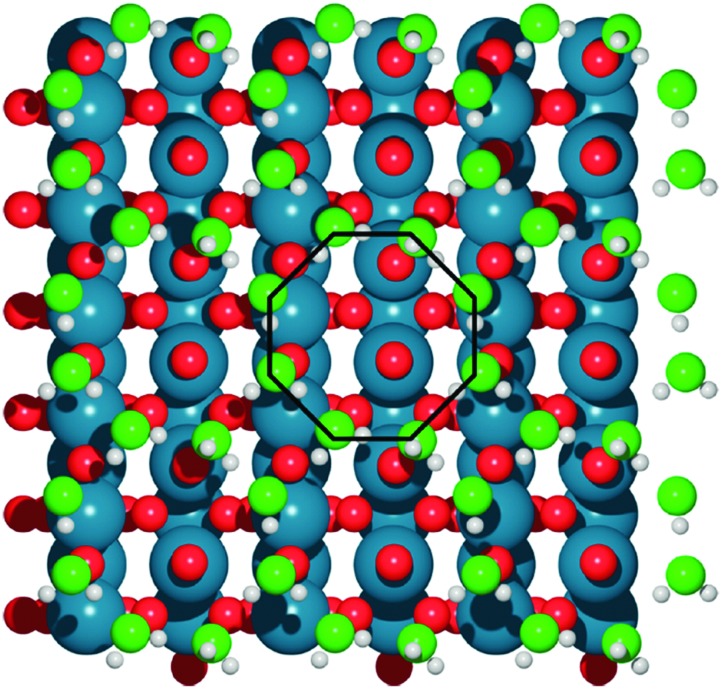
The optimal, octahedral structure of a water bilayer upon IrO_2_, determined with DFT minima-hopping. Water oxygen atoms are light green, surface oxygen atoms are red, iridium is blue and hydrogen is white. Reprinted with permission from ref. 18. Copyright 2017 American Chemical Society.

Basin-hopping (BH)^14^ is a Monte Carlo based global optimization technique, and has a long history of application to materials science. This method belongs to a class of energy landscape-simplifying hypersurface deformation techniques, which remove barriers to energetically downhill steps, and vastly improve the ergodicity of the exploration. Extensive modifications to the original BH method have been developed since the late 1990s. One of the most notable examples is minima hopping (MH), from Goedecker,^15^ which applies short bursts of molecular dynamics (MD) simulations between local optimization steps, with a variable temperature parameter to allow for escape from deep basins. This technique has been widely used, for example, in GO for large gold clusters (up to Au_318_),^16^ the discovery of a new, photocatalytically promising titania nanosheet isomer^17^ and in determining the role of solvating water in electrochemical water oxidation catalysis over IrO_2_(110) (see Fig. 2).^18^ Sicher found that the MD moves in MH are more efficient than saddle point search methods for escaping minima, requiring fewer force calculations to achieve the same success rate.^19^,^20^ Another development is the parallel excitable walkers (PEW) method of Rossi,^21^ which combines a modified tabu search to avoid stagnation in previously visited basins, with the benefits of multiple simultaneous searches. The walkers move in parallel on the same energy landscape and avoid sampling the same region of configuration space by dynamically repelling each other. Walkers are determined to be neighbours based on an order parameter for structural similarity. If two walkers are too close together, the Metropolis ratio is shifted to allow for more unfavourable uphill steps to be accepted. The advantage of the method over traditional tabu sampling is that isolated walkers retain the sampling efficiency of basin hopping, without wasting cpu time rejecting steps due to fixed energy penalties. As an example, Ferrando and coworkers have applied this method for the geometry optimization of binary transition metal nanoalloy clusters.^21^,^22^

Nature inspired GO methods have also been extensively applied to catalytically relevant systems. These methods include, but are not limited to ant colony, artificial bee colony and particle swarm methods.^13^ The most popular class of nature inspired methods by far has been the genetic or evolutionary algorithm (EA).^12^,^23^,^24^ EAs employ a series of steps which mimic changes that occur to a population of individuals in nature, such as mating and mutation.^25^ These steps interchange and modify the structures of a population of trial solutions on a generational basis, until convergence to the putative globally optimal structure. Convergence is defined heuristically, either by a preset choice of generations^26^ or some stagnation criterion in either energies or structures.^23^ Very recently, machine learning techniques have begun to be implemented into the selection step of EAs. Jørgensen *et al.* introduced a clustering method to the EA maintained within the atomic simulation environment (ASE).^27^ This technique groups together elements of the population into classes based on a structural similarity metric. The selection of the next generation of structures is thus biased to focus on promising regions of configuration space. Two algorithms have been tested: one in which unexplored structural classes are biased towards, and one in which over-explored regions are biased against. Clustering was successfully applied both to gas phase molecules and the catalytically important anatase TiO_2_(001) surface.

In both Monte Carlo and nature-inspired GO strategies, the choice of moveclass for exploring the energy landscape is crucial to the efficiency of the search, and much of the improvement of these methods comes from designing and combining effective moveclasses. For example, SHAKE moves^28^ for both EA and BH methods involve moving all atoms by some fixed amount. A variant of the SHAKE move was introduced by Kim *et al.*,^29^ in which each atom is moved during the BH step, but within a displacement range that is a function of the distance of the atom from the centre of the system. This modification mimics the increased diffusivity of atoms near or at the surface of a particle, and was found to enhance the efficiency of the GO search by a factor of 3.8 over standard BH for an AuPd cluster. Continuous symmetrisation techniques have been developed by several groups,^30^,^31^ which improve search efficiency by biasing moves towards the completion of high symmetry structures. Schönborn *et al.* have reported the effectiveness of an “average offspring” EA mating method.^32^ In this strategy, two parents are selected. For each atom of the first parent, the position of the nearest atom in the second parent is located, and the child is assigned an atom at the average of those positions. More recently, Vegge used radial cuts to improve the search for binary particles which show a tendency towards core–shell structures.^33^ The optimal choice of moveclass is thus arrived at heuristically, but allows for flexibility in the GO method, to treat many types of system efficiently.

Moving beyond simply the location of the global minimum, pathway sampling methods combine discovery of the low-lying regions of the energy landscape with identification of paths that connect the minima together. In this way, one can move from static structure prediction to estimation of chemical properties. As with GO methods, the automated nature of path sampling allows for the avoidance of the biases of prior human intuition. Much work has gone into improving path sampling methods in two main ways. First are the algorithms which connect minima, such as eigenvector-following to follow soft-mode pathways, and their hybrid implementations.^13^,^34^ Nudged elastic band (NEB) methods also belong to this group,^35^ as do string methods.^36^–^38^ Second, there are the global searches which utilise these minimum-connecting steps. Discrete path sampling is one example,^13^,^39^ in which single or double ended path searches aim to find and connect adjacent minima and build up a picture of the energy landscape in an automatic manner. These search methods have even been applied beyond catalysis, for example, in recent work on the migration of lithium cations in the Li_0.5_MnO_2_ battery materials.^40^ Another related example is the minima hopping guided pathway approach of Schäfer *et al.*^41^ The stochastic surface walking method of Zhang *et al.* is a promising variant which was designed specifically for chemical reactions.^42^ Connections between minima which are defined as reactants and products according to criteria such as bond connectivity are discovered so as to target promising reaction paths. The search mode may be biased according to particular reaction coordinates to speed up the search. This method has been recently applied to the water gas shift reaction on Cu(111), isolating a new mechanism for formic acid formation.

### 2.3. Free-standing particles

The simplest model for a catalytic nanoparticle is that of a free-standing cluster in a vacuum. This approximation is reasonable either as a first order interpretation of the particle under inert atmospheres, or under the assumption of ultrasoft landing on inert supports. Of course, in most applications, the role of surface, solvation and ligands is important. Nevertheless, a great deal can be learned from knowledge of the geometric and electronic structure of vacuum-phase clusters, and this has traditionally been the starting point for nano-catalysis GO.

Early studies investigated the structures of model particles on the order of 100 atoms, utilising a range of empirically parameterized potentials (EP), such as Gupta^43^,^44^ and others.^16^,^45^ Focussing mainly on the structural motifs favoured by various mono- and bimetallic clusters, these studies revealed a complex landscape of preferred structures in the non-scalable size regime from sub-nm to a few nm, including disordered morphologies, polyicosahedra, prolate disk-like structures^44^,^46^ and strained, even chiral particles.^47^ Increasing computational resources and the development of robust density functional software packages have allowed for electronic structures to be seriously investigated in GO methods. Two phase optimization techniques, in which a pre-screening global optimization is undertaken at the forcefield level, followed by a reoptimization of the promising structures at the DFT level have become common.^48^ However, the risk of the two phase approach is clear: those structures which are preferred with DFT, but not at the forcefield level, are screened out and lost. One way to minimise this effect is to parameterise a forcefield against DFT data. Such parameterisation has been applied extensively by Johnston and coworkers, for bimetallic clusters such as Au–Pd^49^ and Cu–Ag.^50^ For ultrasmall vacuum-phase model catalytic particles, the small size both requires and allows for GO with more accurate, electronic structure methods. The most well studied class of systems is that of gold, and doped gold clusters,^51^–^54^ for which the high degree of relativistic s–d hybridisation is key. DFT-GO has even been used in conjunction with TD-DFT and ion mobility simulations to fingerprint isomers in a cluster beam.^55^ In such an area, where particles exist transiently, or are difficult to isolate, DFT-GO can provide support. The additional complexity of the energy landscape for multicomponent cage systems necessitates an unbiased exploration of configuration space, such as the DFT tabu search for cationic Cu–Sn core–shell clusters^56^ and the DFT-EA approach used for Bi–Sn cages.^57^ For transition metals, the complex spin arrangements are difficult to predict, owing to the subtle balance between the magnetic moment and structure. As an example, consider ultrasmall Ru–Sn particles. Sn-Doping into noble metal particles is known to enhance catalytic activity, while reducing the manufacturing cost, by replacing some of the expensive platinum-group metal.^58^ Paz-Borbòn and coworkers investigated the properties of Ru_2*n*_, Sn_2*n*_ and (RuSn)_*n*_ clusters (*n* ≤ 6) towards the catalytic hydrogenation of ethylene with a DFT-BH approach, combined with NEB to determine reaction barriers.^59^ They observed that the inclusion of tin drives a profound change in the structure, from cubic towards compact, Sn-capped structures, and a reduction in the total magnetic moment. These changes coincide with a decrease in the rate-determining step barrier, and thus an enhancement of reactivity, in agreement with experimental findings. An interesting development, which combines DFT-GO with *ab initio* constrained thermodynamics, was made by Scheffler *et al.* to isolate the global minima of clusters at finite temperatures in the presence of oxygen.^60^,^61^ This method was applied to Mg_*x*_O_*y*_ clusters over a range of sizes (*M* < 16), spin states and stoichiometries, finding a surprising preference for non-stoichiometric particles at small sizes.

#### 2.3.1. Ligand-passivated particles

Another possible method to stabilize (sub)nanometer clusters and control their size distribution is to passivate them with (organic) ligands.^10^ Passivation of the cluster can lead to two types of cluster–ligand complexes depending on the strength of the cluster–ligand interaction and cluster/ligand concentration:^62^ (i) a simple association complex of the ligand with the cluster's global minimum (GM) (or few low-energy isomers), or (ii) a cluster–ligand complex with weak topological similarities to the cluster's gas-phase GM. The former type is amenable to a two-phase GO procedure. In this procedure, low-energy cluster isomers from the gas-phase are obtained first, followed by optimization of the position and orientation of the ligands, which decorate the cluster core. For the latter cluster–ligand type, often full GO of cluster–ligand species is necessary. Both types of cluster–ligand and GO approaches have been considered in GO studies of passivated clusters. These studies are dominated by two classes of systems, the thiolate-protected gold^63^–^65^ and silver^25^,^66^–^68^ clusters and the hydrogen-passivated silicon clusters.^24^,^50^,^69^–^73^

**Fig. 3:**
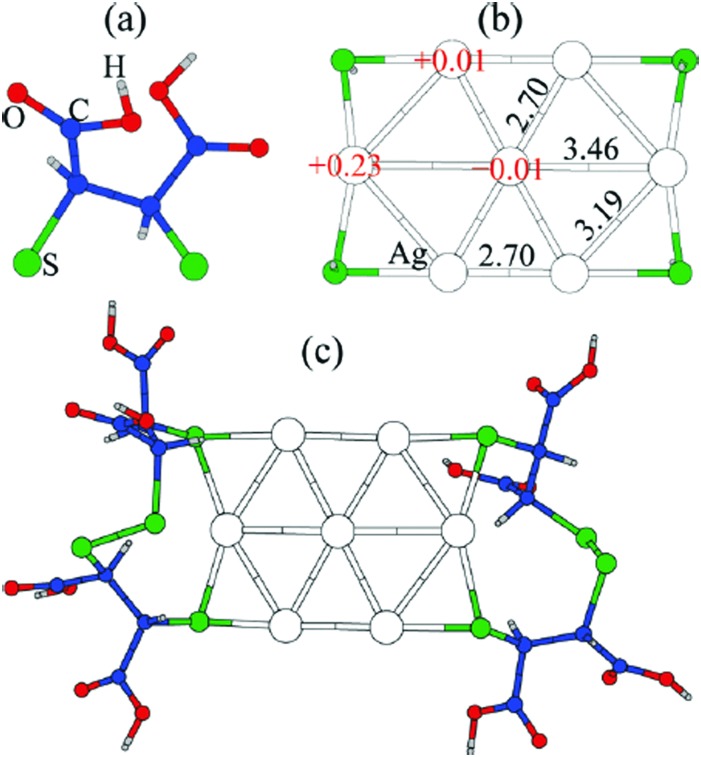
Structure of the GM for Ag_7_(DMSA)_4_^–^ determined with the GA of Xiang *et al.* (a) the structure of the DMSA ligand. (b) The GM determined with minimal sulphur capping atoms, showing Bader charges and Ag–Ag bond lengths, (c) the complete structure of the complex. Reprinted with permission from ref. 66. Copyright 2010, American Chemical Society.

Thiolate-protected gold clusters have been used as a model system for metal nanoparticles because of their extraordinary stability^74^ and availability of synthetic strategies able to prepare monodisperse clusters in high yields.^75^,^76^ The interest peaked with crystal structure determination of Au_102_(SR)_44_^77^ and Au_25_(SR)_18_^78^ clusters, which were formed from a high-symmetry Au core capped by “staple” motifs -RS-(Au-RS)_*n*_-Au-RS- (*n* = 0, 1). This “divide and protect” structural concept,^79^*i.e.* division of the cluster into the metal core and protecting ligands, has been utilized in the first two-phase GO studies^63^,^64^ on Au_20_(SR)_16_ and Au_24_(SR)_20_. In these studies Pei *et al.* employed EP-driven BH for an Au core supplemented by manual construction of ligand protections of various lengths that still fulfill the constraints of the molecular formula, which was then followed by local DFT optimizations of the assembled Au_*n*_(SR)_*m*_ cluster. The “divide and protect” concept influenced also the first direct GA-based DFT-GO by Xiang *et al.*,^66^ which they applied to the Ag^7–^ cluster, ligated with (SCH_3_)_4_ or (DMSA)_4_. Their procedure involves performing mating and mutation steps on the metal cluster core, with only one ligand atom (sulphur) bound at the core surface, followed by the re-introduction of the remaining ligand chain for local geometry optimization, as depicted in Fig. 3. Before carrying out the local DFT optimization, a fast EP-based Monte Carlo run is used to reorient ligand chains to minimize their steric repulsion. Recently, a full DFT EA-GO of cluster–ligand species has been employed^65^ to search for structures of (AuL)_*n*_ (L = Cl, SH, SCH_3_, PH_2_, P(CH_3_)_2_, *n* = 1–13) clusters. The high ligand concentration (Au-to-L ratio 1 : 1) in these structures prevents formation of an Au core both invalidating the “divide and protect” concept for these stoichiometries and justifying the use of standard cluster GO implementation without any passivation-specific improvements/biases. Lastly, in a number of studies, Bonačić-Koutecký *et al.*^25^,^67^,^68^ investigated thiolate-protected silver clusters using simulated annealing at the semi-empirical AM1 level to obtain candidate structures for the subsequent local DFT re-optimization. In their works, the authors illustrated how increasing ligand concentration lengthens the staple motifs -RS-(Ag-RS)_*n*_-Ag-RS- until the whole Ag core is consumed and all that is left are the differently interconnected (Ag-RS) units.^25^

The second class of cluster–ligand systems investigated using GO methods are silicon nanoparticles, passivated with hydrogen. The justification for the interest in these systems comes from their potential use in optoelectronics, solar cells or photocatalysis, stemming from their strong size-dependent photoluminescence, photostability and the possibility of preparing highly monodisperse systems.^33^,^80^ In addition, the biocompatibility of silicon and its flexible surface chemistry that facilitate water dispersibility and easy conjugation of DNA or protein probes have made these systems intriguing for bio-imaging or for use as biosensors.^81^ The pioneering work on GO of hydrogen-passivated silicon clusters has been done by Ge and Head who in a sequence of studies^24^,^69^–^71^ gradually perfected their EA-based GO implementation. In their first study,^69^ a standard two-phase GO was used, employing the AM1 method to locate several low-lying structures of Si_10_H_*n*_ (*n* = 4, 8, 2, 16, 20) and Si_14_H_20_, followed by re-optimization at the MP2 and DFT levels. For large, well-passivated Si_14_H_20_ clusters, the emergence of the bulk Si diamond-lattice structure as GM was observed. The only passivation-specific modification to standard cluster EA-GO involves generation of the initial population; first, the randomly generated Si core was created inside the cubic box and then the H atoms were randomly arranged near the box borderline, either outside or inside. In the follow-up study,^70^ the authors tried to mitigate the incorrect AM1 energy ranking by proposing an iterative GO strategy involving two separate EAs invoked consecutively. One is the standard cluster EA (CEA) used for structure optimization at the AM1 level, while a second EA is used to reparametrize the AM1 method using a growing set of reference *ab initio* data (either DFT or MP2) obtained from local *ab initio* re-optimization of low-energy isomers from previous CEA runs. The two separate EAs are performed iteratively until the AM1 parameters give an energy ordering that is consistent with the accumulated *ab initio* database. Although such adaptive, on-the-fly re-parametrizations tailored for a specific problem at hand hold, in our opinion, great potential for the future (with availability of fast computers and robust fitting approaches^82^) (see Section 6 on machine learning approaches), in the early 2000s this approach was deemed prohibitively expensive^70^ and was not pursued further. Rather, a fixed set of improved AM1 parameters, termed the GAM1 method, was obtained^24^ from the Si_7_H_14_ training set, and considered transferable to other Si_*n*_H_*m*_ stoichiometries. The sequence of studies by Ge and Head concluded^71^ with the introduction of new system-specific mutation operators such as SiH_3_ removal, SiH_2_ removal or H shift. New mutation operators combined with the previously re-parametrized^24^ AM1 method improved convergence of GA, in particular for problematic cases with Si_14_H_20_ and Si_6_H_6_ stoichiometries. Recently, small H-passivated silicon clusters have also become the subject of a direct DFT EA-based GO investigation by Baturin *et al.*^50^ using a cluster EA implementation in the USPEX code.^83^ This study of Si_10_H_2*m*_ (*m* = 0–12) nanoclusters highlighted how hydrogen concentration, temperature and density of low-energy isomers affect the structural and compositional flexibility of the nanocluster ensemble, possibly making experimentally realizable cluster compositions highly non-uniform, both structurally and compositionally. Indeed, with increasing size of the cluster in question, the number of low-energy isomers explodes, making the comprehensive search for configurational space prohibitively expensive. Rather, it becomes necessary to employ strategies capable of obtaining representative structures under given experimental conditions (*e.g.*, concentration of the reactive compounds) with topological properties (*e.g.*, concentration of defects) consistent with experiment. Some work in this direction was done by Biswas *et al.*^72^,^73^ who used EP-driven metadynamics simulations supplemented with simulated annealing runs at the non-self-consistent DFT level using the Harris functional^84^ to obtain models of hydrogenated amorphous silicon (a-Si:H). Rather than obtaining the converged free-energy surface of such a large and complex system, the purpose of the metadynamics simulations in this study was to generate configurations with specific topological properties (*e.g.*, Si dangling-bond defects), which are consistent with experimental data from IR and NMR spectroscopies. In particular, the EP-driven metadynamics run, using the average coordination number of silicon atoms as a collective variable, produced an ensemble of a-Si structures with a defined number of undercoordinated Si atoms, which were passivated by hydrogen atoms, using a simple geometric construction to achieve maximal tetrahedral character of defective Si sites, and re-optimized at the non-self-consistent DFT level.

The final class of passivated clusters investigated at the GO level are the hydroxylated silica (silicon dioxide) clusters, a system of ubiquitous fundamental importance (*e.g.*, in mineral nucleation, growth and dissolution processes or in synthesis of nanoporous silicate materials such as zeolites), investigated by Bromley *et al.*^85^,^86^ In their first study,^85^ a standard two-level BH-GO scheme was employed, in which several tentative low-energy isomers of (SiO)_*n*_(H_2_O)_*m*_ (*n* = 4, 8, 16, 24 and *m* up to *m/n* ≥ 0.5) obtained at the EP level were re-optimized using DFT. In the follow-up study^86^ on (SiO)_*n*_(H_2_O)_*m*_ (*n* = 6, 8, 10, 12), the authors refined their BH-GO approach by employing a two-step local optimization in each BH step, termed a cascade basin hopping approach, where first a simple and computationally efficient EP is used to pre-optimize the new distorted structural candidate, followed by a more sophisticated EP to carry out full relaxation accounting for polarization and H-bonding. These GO investigations managed to capture two very distinct structural regimes in the (SiO)_*n*_(H_2_O)_*m*_ system – while small clusters are progressively hydroxylated with increasing water content, the larger clusters tend to form dense amorphous clusters with hydrogen-bonded surface water molecules. This highlights the importance of un-biased (global) structure optimization approaches to correctly predict the structures of passivated clusters as a function of the cluster size or passivation degree.

### 2.4. Structures of catalyst surfaces

Extended exposed surfaces, which are crucial to heterogeneous catalysis, are usually more geometrically restricted than free particles, owing to the periodicity of the crystal and the presence of strong covalent bonds to the bulk below. As a result, GO for surfaces has received less attention computationally than isolated particles, with simplified periodic models deemed to be sufficient for most investigations. Such models are, however, problematic in exceptional cases. These cases include thin films, where the surface layer may be structurally and electronically distinct from the bulk, due, for example, to incomplete growth or undercoordination. In this area, GO methods are useful in combination with surface science experiments. Another case is for real catalysts, in which complex physicochemical conditions are present at the surface. Oxygen pressures, access to potential adsorbates and temperature can all drive dramatic changes to the surface layer. Metal oxide surfaces, which are often involved in oxidation, photo- and electrocatalysis, are particularly prone to such effects, and have received increased attention recently.

Development of GO methods for periodic condensed matter includes the periodic cut,^87^ which is useful for combining parent structures for EA mating steps in systems with periodic boundary conditions and different supercells. Another improvement is the mating slab procedure of Chuang *et al.*,^41^ in which a minority section at the top of the slab is chosen to mate between elements in the population, keeping the bulk-like layers below, fixed. The cutting plane was found to be optimal when unconstrained to pass through the cell centre, and kept away from the cell boundaries. The approach was applied for an illustrative example of silicon.

In order to support surface science experiments in the elucidation of complex surface phases, DFT-GO has developed as a useful characterisation tool. In an early example of DFT-GO, Sierka *et al.* determined the stable geometries of oxidised Mo(112) in the p(1 × 2) and p(1 × 3) structures^88^ with an evolutionary algorithm, called the hybrid *ab initio* genetic algorithm (HAGA).^89^ An oxygen-induced missing-row reconstruction was observed in both cases, which coexist over a wide range of oxygen partial pressures. Later work applied the same method towards the more complex O(2 × 3)–Mo(112) system,^90^ again finding better agreement with experimental data than previous models. The HAGA method is similar to other EAs except that it can be used in a constant chemical potential mode, rather than the standard constant composition mode. This allows for the fitness determination of elements of the generation to be ranked by the approximate free energy of formation of the product state, rather than the total internal energy. This is beneficial when studying the chemical reactions that form oxide surfaces. For MoO_*x*_ surfaces, the relevant reaction is the formation of the metal oxide from the Mo(112) surface and molecular oxygen. Evolutionary algorithms continue to be used to elucidate structures of reactive oxide surfaces which have eluded experimental characterisation. The structure of the 4 × 1 reconstruction of SnO_2_(110), which is active in oxidation catalysis of CO and of CO/NO, and as an activity-enhancing support for metal particles,^91^ has been unknown since the 1980s. It was very recently determined, using a combination of DFT-GO and experimental surface X-ray diffraction by Merte *et al.*^92^ The surface is found to be terminated by an ordered array of Sn_3_O_3_ clusters upon the bulk termination of SnO_2_(110).

For complex catalytic surfaces under real conditions, defects, such as steps, vacancies and adgrowths are common. In these cases, GO may still provide insight. This is the case for the prototypical TiO_2_(110) surface, which is an important and well-studied system for photocatalytic oxidation reactions.^93^ Martinez *et al.* employed a DFT-EA to explore the local structure of the common employed a DFT-EA to explore the local structure of the common 〈11̄1〉 and 〈001〉 step edges of TiO11[combining macron]1 employed a DFT-EA to explore the local structure of the common 〈11̄1〉 and 〈001〉 step edges of TiO and employed a DFT-EA to explore the local structure of the common 〈11̄1〉 and 〈001〉 step edges of TiO001 employed a DFT-EA to explore the local structure of the common 〈11̄1〉 and 〈001〉 step edges of TiO step edges of TiO_2_.^94^,^95^ The authors found new step edge structures which are more stable than the bulk termination, and the presence of O vacancies, which are active in ethanol dissociation. Bechstein *et al.* applied a DFT-EA to explain the presence of reduced strand-like Ti_*x*_O_*y*_ adgrowths at TiO_2_〈11̄1〉 step edges, which are observed in STM images.11[combining macron]1〈11̄1〉 step edges, which are observed in STM images. step edges, which are observed in STM images.^96^ By separating the strand, which is around 6 nm in length, into distinct regions (the connection region, the strand region and the end-of-strand region), they could unravel the structure of a large system, considering three separate global optimization investigations in parallel. In this way, the unbiased optimization of the local structure allowed for geometries to be discovered which are unexpected from prior chemical intuition, or which would be implausible to otherwise study, due to the vast configuration space available.

Surface GO methods are not only used in support of existing experiments. Theoretical investigations on surface structure have occasionally predicted stable phases of materials before experiment. An MH-GO study on free-standing TiO_2_ nanosheets has recently predicted a new honeycomb isomer that is lower in energy than those previously discovered.^17^ Using an artificial neural network potential that was trained on DFT structures, the novel isomer was determined to have a good band alignment with the redox potential for water splitting, and thus is promising from the point of view of both synthesis and catalysis. 2D confining potentials were employed, which is a general procedure for 2D material GO. Randomness was maintained in the search path out of basins by choosing a soft mode, while relaxing the constraint to adopt only the lowest frequency eigenmode. In fact, this choice is the reason MH is found to be more efficient than saddle-point methods.^32^ The latter methods either take the lowest mode, which is not guaranteed to correspond to a low energy path, or calculate paths along all eigenmodes, which is computationally demanding. For ultrathin films of AlN, BeO, GaN, SiC, ZnO, and ZnS, DFT calculations have suggested a graphitic structure for the thinnest films of each species, which convert to the polar (0001)/(0001[combining macron]) above a certain number of layers, and are stabilised by charge transfer.^97^ In a similar spirit, the simulated mechanical annealing method of Bromley and colleagues has been used to predict novel phases of catalytically important reduced cerium oxide surfaces,^98^ ZnS nanosheets^99^ and nanotubes.^100^ The approach involves gradually compressing and expanding the system, locally relaxing the geometry at each point, and capturing any new local minima. The process is repeated for each new minimum until the structural space is exhausted.

### 2.5. Surface-deposited particles

In heterogeneous catalytic experiments and industrial applications, active catalysts are often made up of clusters and nanoparticles supported by stable, insulating surfaces. The role of the surface is much more complex than simply providing additional physisorption to stabilise the particle against sintering.^71^,^101^ Major factors which must be present in GO investigations to treat surface-supported particles include: (i) the effects of lattice strain and epitaxy with the surface, and any particular effects of strong adsorption, (ii) charge transfer between surface and particle, defects and the possibility of sintering and particle migration, (iii) the effect of solvent, adsorbates and relevant reactions on the catalyst structure, and (iv) encapsulation, for catalytic particles contained within a confining environment. Examples of each issue are considered in the following section.

#### 2.5.1. Strain, epitaxy and adsorption strength

Miyazaki and Inoue probed the effects of tuning the interaction strength between a cluster and a support with an early surface genetic algorithm.^102^ Binary strings encoded the structural information, within a lattice model for discretizing space. The cluster atom size and relative strength of intra-particle and particle–surface interactions were modelled with a Lennard Jones potential. Particles which adopt icosahedral morphologies in the vacuum phase were found to wet the surface, forming either monolayer islands or condensed layered structures, depending on the interaction potential. For large cluster atoms, the potential well for cluster–surface bonding was narrow, and thus smeared out, inducing full surface wetting. This is an early example of lattice effects being directly responsible for cluster structure in GO investigations. In another early surface EA-GO study, Zhuang *et al.* determined the global minima of adatom clusters Al_*n*_, Ni_*n*_, Ag_*n*_, Pd_*n*_ and Pt_*n*_ (*n* ≤ 40) upon (111) surfaces,^103^ with modified embedded atom potentials. Clusters generally favoured structures which maximised the number of nearest neighbours, but discrepancies were found for systems where the adatom–surface interaction was particularly strong, allowing for nearest neighbour bond-breaking to be compensated for by strong adsorption of edge sites to the surface. Recently, Eckhoff and colleagues extended the analysis of adsorption to generic pristine surfaces, focussing on the mechanical properties of the surface, and their effect in driving the geometry of the particle.^104^ They report that the surface microstructure, defined by the lateral strain of the substrate, can have profound effects on the preferred cluster morphology on pristine supports. Stacking faults, twinning and reorientation of the cluster can all be observed in global minimum energy structures, along with reordering of the relative stability of structural motifs. It should be concluded that lattice mismatch and strain is sufficient to access the full range of possible adsorbate structures, even in the absence of surface roughness or defects. Lattice mismatch between the adsorbate and surface is important in the growth, structure and stability of particles, and has been studied in detail with two phase EP/DFT-GO methods for model surfaces and metal particles.^105^,^106^ For cubic lattices, such as MgO(100), cubic phases develop in the adsorbed particle, which modify the facets exposed to potential gas phase reactants. The balance between surface epitaxy and the natural preference for close packed structures undergoes a crossover at a certain size. Goniakowski *et al.* used BH and PEW for noble and coinage metal clusters of selected sizes up to 500 atoms on MgO(100).^106^–^108^ A size-dependent transition from cube-on-cube (100) structures to fcc (111) motifs was found. The onset size of the transition was observed to be smaller for particles with larger lattice mismatch with the surface.

For multicomponent particles, the different strength of surface adsorption for the component elements can drive surface-induced segregation, and even affect the preferred structure of the cluster. For example, Ismail *et al.* used a basin hopping algorithm with a two phase EP/DFT-GO approach to investigate the segregation of Pd to the surface, for adsorbed AuPd clusters on MgO(100).^105^ Exchange moves were employed at a frequency of 10% to speed up the search for the wide permutational isomer space. This is a crucial consideration for the efficiency of GO methods on multicomponent systems. In both Monte Carlo and nature-inspired methods, the choice of swap move frequency is made, based on the balance between the structure and permutational isomer optimization.

Subnanometre-scale metal particles on oxide supports have been intensively investigated, especially since the discovery of enhanced intrinsic catalytic activity at ultrasmall sizes. Sub-nanometre sized metal particles have been shown to exhibit even higher activities in heterogeneous catalysis (Cu for CO-to-methanol conversion,^109^ Ag for propylene epoxidation,^110^ Au for alkyne hydration,^74^ Pd for electrocatalytic water splitting^87^ and Pt for propane oxidative dehydrogenation^111^). As in the case of ultrasmall isolated particles, the GO of the very smallest particles upon surfaces requires the accurate capture of electronic properties, such as the “metal-on-top effect”^112^ and spin effects. Davis *et al.* used the recently developed “pool EA” to examine the stabilisation of Au subnanometre particles on MgO(100), by alloying with iridium. The authors found significant stabilisation of the particles as the iridium content was increased, in agreement with experiments, which suggested an enhanced sintering resistance.^113^ Vilhelmsen and Hammer developed and applied a direct surface DFT-EA for subnanometre clusters of gold upon MgO,^114^ for which the upper surface layers were free to locally relax during local optimization. They found a number of Au_8_ structures lower in energy than previously reported, despite the great number of studies performed without unbiased GO methods. This is a clear indication of the need for an open-ended GO search. More recently, the same group has developed and benchmarked their EA, which is built into the atomic simulation environment (ASE), to more efficiently perform direct DFT-GO.^26^ The authors introduced a series of new moveclasses, specific to adsorbates upon a surface. One such moveclass is the rotation mutation, which moves the cluster around the surface normal, so as to locate the optimal overlap between the cluster and surface.

**Fig. 4:**
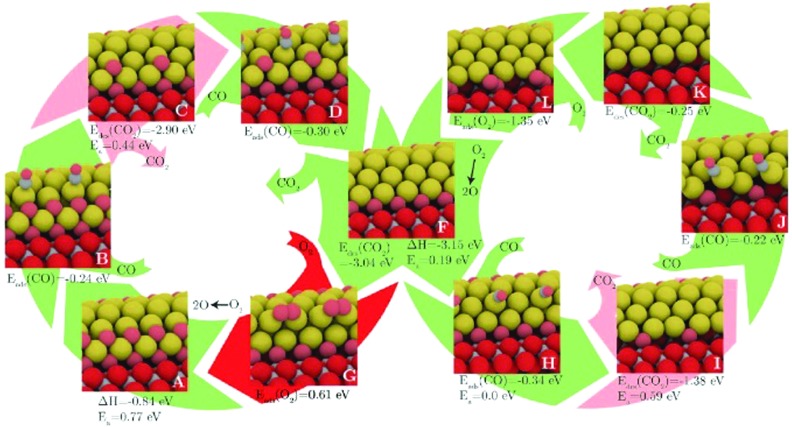
The structures and energetics of CO adsorption, CO_2_ production and subsequent reoxidation of the (100)-oriented Au nanorod edge on TiO_2_(110). Barriers for CO_2_ formation are prohibitively high, while the reoxidation of the reduced gold nanorod is facile. Reprinted with permission from ref. 115. Copyright 2013 American Institute of Physics.

Another is the symmetrisation operator, which reflects half of the cluster across a mirror plane drawn through its centre. These moves yielded only small improvements to the search efficiency, but it should be noted that the GM was not difficult to locate, with a success rate of around 98%. Hence the increase in diversity introduced by the new steps was probably not necessary in the tested case. One may expect that for a more difficult case, these moves would have a more significant effect. Extended to a (100)-oriented Au nanorod upon TiO_2_(110), the authors predict an unusual interfacial layer of oxygen, which was unlikely to be predicted from a biased search.^115^ The thermodynamics and kinetics of CO oxidation on this system were subsequently examined, as depicted in Fig. 4. DFT-BH has also been applied to subnanometre gold clusters on hydroxide supports^116^,^117^ which are proposed to exhibit good low temperature CO oxidation activity.^118^ The activity is explained by charge transfer between the surface and the gold cluster, which activates the O–O bond of adsorbed oxygen.

#### 2.5.2. Charge transfer, sintering and migration

Particle sintering is a major deactivation route for industrial catalysts. Defects at surfaces play a large role in trapping catalytically active species, and are important in understanding the processes of growth and coalescence. Defects are often unavoidable consequences of surface preparation, but can also be caused by the adsorption of catalytic particles, through charge transfer and surface abstraction. However important, the inclusion of defects adds no conceptual complication to GO methods, and so only some interesting results will be discussed here.

Some of the first full DFT-GO studies of catalytic particles on defective oxides were performed using a DFT-BH method to investigate Ag and AgPd clusters on MgO(100). On the neutral double vacancy, which is common in MgO preparation, the recovery of the gas phase Ag_8_ magic number cluster was predicted.^82^ This cluster exhibits large HOMO–LUMO gaps and stability with respect to nearby sizes. By contrast, a DFT-BH investigation by the same authors on Ag_*n*_ (*n* ≤ 11) on the F_s_ vacancy of the same oxide shows the complete loss of the magic number.^119^ The frustration between the metal–metal bonding and the distortion necessary to maximise the Ag-vacancy interaction leads to a global minimum structure which is distorted and not particularly stable. The apparently complex interaction between catalytic particles and the defective support is based on the balance between satisfying the closed-shell stable structures found in the vacuum phase and maximising bonding to the TiO_2_ (110) surface. This balance is difficult to predict without a full GO investigation. Another important catalytic system is that of Pt upon ceria surfaces, which are used in automotive catalysis and have been recently proposed as a potential high power density PEM fuel cell anode.^120^,^121^ Paz-Borbòn and colleagues recently employed a DFT surface GO method to determine the low energy structures of Pt_*n*_ on a CeO_2_(111) surface (*n* ≤ 11).^122^ Basin hopping coupled to a plane-wave DFT code allowed the authors to locate putative global minima across the size range. It was found that 2D Pt structures are preferred up to *n* = 8, owing to the strong interaction between the metal and substrate. Charge transfer occurs from Pt to surface oxygen, along with reduction of Ce^4+^ to Ce^3+^. This is in good agreement with studies which show the reducibility of ceria to pin particles to the surface through charge transfer.^123^ Experimental data suggest that charge transfer goes through a per-atom maximum for particles of around 50 atoms.

The development of ultrahigh vacuum deposition techniques and the sophisticated surface science characterisation methods have raised the questions of trapping, migration and coalescence of catalytically relevant deposited particles.^124^ The diffusion pathways of ultrasmall particles have been studied computationally mostly with pathway search methods as described in Section 2.1. Upon the model metal oxide MgO(100), Xu *et al.* calculated the diffusion pathways and rates according to harmonic transition state theory.^125^ Interestingly, they observed that tetramers were even more mobile than monomers, while single atoms are not especially attracted to Pd_1_/F_s_ sites. Hence, the sintering mechanism was predicted to be one of Pd atoms trapped at defects, while small clusters grow and freely migrate around the surface until coalescence with the Pd_1_/F_s_ centres. This differs from the previous model of single atoms combining with Pd_1_/F_s_ centres and growing in a stepwise fashion. The predictions were further tested with kinetic Monte Carlo simulations over the 200–800 K temperature range, finding excellent agreement with experiment.^126^ Similar findings have been made for coinage metal clusters,^127^ suggesting the importance of small particles in coalescence processes. The vast configuration space available to particles larger than 1 nm makes global searches for migration and sintering pathways prohibitively expensive, though studies have aimed to describe the kinetics of Ostwald ripening processes by fitting DFT energetics to sintering rate equations.^128^

#### 2.5.3. Adsorbates and reactions

As is clear from the range of elements, surfaces and applications of the above studies, the use of global optimization techniques for sub-nanometre sized catalysts on supports has become relatively standard, both as an unconstrained investigative tool and for supporting experimental characterisation of complex systems. The methods are robust, and the application of the appropriate model is becoming ever more important. The model should ideally include all present species, both reactive and inactive, to represent the correct environment of the real catalyst.

It was found by Wang and Hammer that under the reduced conditions relevant for surface science experiments, the Au_7_ cluster is only weakly adsorbed to the surface.^129^ By contrast, under the oxidising conditions of the real catalyst there is strong adsorption of partially cationic gold, which leads to low CO oxidation barriers. Hence, the problem of transferring results between different experimental techniques is also a concern for the choice of model in GO studies. The adsorption and diffusion of Pt clusters on TiO_2_(110) surfaces^130^ has also been studied with a DFT-EA approach. Oxidised (O atoms on top), reduced (hydrogen adatoms) and surfaces containing oxygen vacancies were considered in the surface model. These models gave differing results, in good agreement with experiment, that O defects trap small particles, reducing diffusivity and maintaining small particle sizes, while hydrogen has little effect, allowing migration and sintering. While not strictly a catalytic system, the role of solvating water in the stabilisation of particular surface terminations has been probed with a direct DFT-MH investigation for CaF_2._^131^ It was found that the polar (100) termination becomes preferred over (111) in the presence of water, while facile reconstructions between nearly-isoenergetic local minima lead to a fluxional surface structure. In this case, the presence of solvating water is completely responsible for the structure, and the consequent chemistry of the surface.

As stated at the start of the section, the energetics derived from global optimization studies are primarily potential energies, which represent low temperature behaviour, from which thermodynamic approximations may be made. However, catalysis is often a kinetics-driven process. As such, the 0 K approximation tells only part of the story. A method which combines GO, KMC and path sampling was developed by Fortunelli and coworkers with the intention of application directly to catalysis, and is denoted as Reactive Global Optimization (RGO).^132^,^133^ This method aims to globally seek the combined energy landscape of a catalytic particle, the surface and all gas phase reactants of the catalytic reaction in question. The search is based on the calculation of kinetic prefactors and internal energy barriers of elementary steps. The accessible region of configuration space is deliberately limited by choosing cutoffs in the maximum barrier height, which is analogous to a defined experimental temperature. In brief, the RGO process consists of a cycle in which, (i) a structure is identified, (ii) all neighbouring minima are located by following each eigenvector of the Hessian matrix, (iii) barriers are determined for the connection between adjacent minima, (iv) unfeasible steps are purged, (v) the next structure is selected based on a KMC simulation and (vi) adsorbates are added to the structure. One benefit of the method is that it is inherently parallelisable, as multiple walkers may explore disconnected regions of configuration space concurrently, and share structural information so as to avoid repetition. In this way, RGO is similar to the parallel excitable walkers method. Additionally, by virtue of being a kinetic, rather than a thermodynamic process, it is suited to probing kinetically controlled reactions. Furthermore, complex ligand effects, such as the adsorbate-induced decomposition of small particles may naturally be taken into account. For CO oxidation over Ag_3–*x*_Au_*x*_, Ag_2_Au_1_ was found to have the best balance of reactivity and stability.^133^ The limitation of the method is the great cost of searching even a realistically truncated potential energy surface in a reasonable time. The selection of eigenvector following methods and the accuracy of the saddle point convergence allow for some tunability, but the problem remains that hundreds of relevant local minima may be present under the conditions relevant for experiment.

#### 2.5.4. Encapsulated particles

The encapsulation of clusters into a host matrix represents a natural way to control their size distribution and improve their sintering resistance.^134^–^138^ Besides imposing steric constraints, encapsulation provides an additional handle to tune the properties of clusters by varying the dielectric and charge properties of the confining environment. However, direct cluster GO in confinement is computationally expensive and thus extensive manual construction of possible clusters in confinement followed by local optimization is still a popular method of choice.^139^–^141^ Admittedly, manual construction is well-justified in situations when formation of only ultrasmall clusters (couple of atoms) is possible due to an extreme confinement, which significantly limits the number of isomers to be tested.

A more involved approach is to utilize a two phase EP-GO/DFT approach, employing GO for gas-phase clusters at the EP-level with subsequent local DFT reoptimization of embedded low energy gas-phase isomers. The applicability of this procedure relies on the assumption that confinement neither causes significant reordering of the gas-phase isomer stabilities nor creates entirely new isomers which are not local minima in the gas-phase. This two-phase procedure was used for Pt_13_ clusters in Y zeolite, using iterative metadynamics^142^ calculations to obtain low energy gas-phase Pt_13_ isomers, and for small copper clusters embedded in the ERI zeolite optimized using an EA.^143^

Finally, a few studies have attempted direct cluster GO in confinement. The most prominent examples are the works of Vilhelmsen *et al.*^144^,^145^ who employed their EA-based GO for clusters on surfaces^114^,^146^ to find global minima structures of Pd clusters embedded in UiO-66 MOF and Pd, Au and PdAu clusters in MOF-74. In these works a cluster is subjected to a GO process inside the flexible MOF nanopore. The only change made to the original EA for supported clusters^114^ (see Section 2.5.1) is the way the starting population was generated; employing either a cylindrical coordinate system in the MOF-74 pore or Monte Carlo technique with insertions, deletions, and displacements for UiO-66. The authors show that interaction with the walls of the nanopores, which are composed of aromatic rings and Zn open metal sites, results in significant deformation of the putative gas-phase GMs of Au_8_, Pd_8_ and Au_4_Pd_4_ clusters, with deformation energies above 0.6 eV, supporting the need for an unbiased GO process. The authors also note that as a by-product of generating a large number of candidate structures distributed through the MOF unit cell during the EA run, a diffusion path from one unit cell to the next can be established through the identified structures. In the follow-up paper on Pd clusters in UiO-66 MOF, much larger clusters were considered (up to Pd_32_). The chosen MOF structure contains pores defined by cages separated by relatively narrow windows (about 3.9 Å), which was hypothesized to stabilize isolated Pd clusters preventing their agglomeration. However, their calculations show that the Pd cluster would not only grow to fill up the cages in the MOF, but interconnect with Pd clusters in neighboring cages to form thermodynamically stable aggregates.

**Fig. 5:**
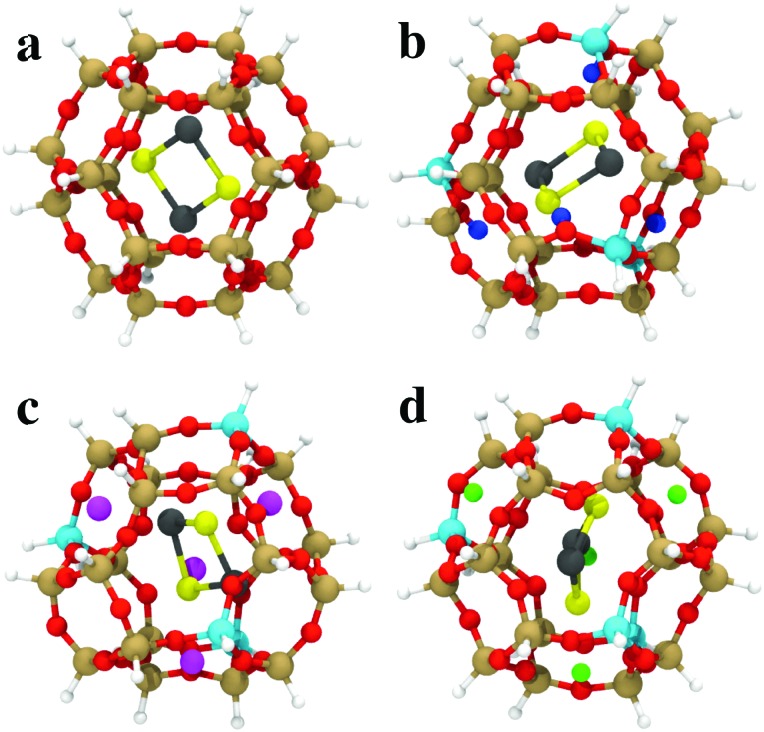
The most stable (PbS)_2_ adsorption configurations in (a) SiSOD, (b) HSOD, (c) LiSOD and (d) NaSOD cages, described by an H-terminated cluster model at the PBE0/TZVP level of DFT. O is red, Si is brown, Al is cyan, Li is magenta, Na is green, Pb is black, S is yellow, terminating-H is white, charge-compensating H^+^ is blue. Reprinted with permission from ref. 147. Copyright 2016, American Chemical Society.

The GA-based GO has also been recently used to obtain the structures of subnanometer (PbS)_*n*_ (*n* < 6) quantum dots confined in a sodalite cage,^147^ which is a building unit of a number of industrially relevant zeolites. The sodalite cage with different compositions (pure silica, H-, Li, and Na-exchanged) was considered and a modified cut-and-splice operator was proposed, which also included parts of the confining environment (extra-framework cations) in the crossover operation. The author reported stability reordering of the isomers with respect to the gas-phase, with changes even to the global minimum. Moreover, these changes were dependent on the type of extra-framework cation present in the SOD cage. Results for encapsulated (PbS)_2_ are shown in Fig. 5. These results hint at the possibility of fine-tuning the structure and properties of embedded clusters with a suitably chosen confining environment. The possibility of tuning the cluster structure by adjusting the environment composition (Al/Si ratio) was investigated also by Palagin *et al.*^148^ in a BH-GO study on subnanometer copper oxide clusters in MOR zeolites. As the copper oxide clusters act as cations that charge-compensate the negative charge of the zeolite framework, the interaction with the zeolite framework is strong, substantiating the need for an appropriate *ab initio* treatment of both the cluster and environment during the GO process. We note that both implementations, the GA-based GO used by Vilhelmsen *et al.*^144^ and BH-GO used in the study by Palagin *et al.*,^148^ are now publicly available through the open source project “Atomic Simulation Environment” (ASE).^149^

Common to all GO methods discussed in this section are the limitations of the harmonic approximation, from which kinetics are derived, and the lack of real temperature effects. The free energy surface, which is the true landscape to be sought, is the subject of techniques such as *ab initio* molecular dynamics and metadynamics, which are considered in other sections of the current review. What these methods gain in accuracy for the calculation of chemical properties, they lack in exploration scope, due to their computational cost. Hence, the use of GO methods for qualitative information, screening of structures and broad comparison to experiment remains valuable in the field of heterogeneous catalysis.

## 3. *Ab initio* constrained thermodynamics

### 3.1. Basic principles

The effect of the finite partial pressures of surrounding gases on the catalyst properties can be taken into account by constrained *ab initio* thermodynamics (AITD) as has been formulated by Reuter and Scheffler.^150^,^151^ The thermodynamic formalism is briefly described below, a more comprehensive review can be found, *e.g.* in ref. 152 and 153 The catalyst environment is described by pressure *p* and temperature *T*. The Gibbs free energy *G*(*T*,*p*,*N*_*i*_*N*_*j*_) describing the system depends on the number of *i* and *j* atoms (*e.g.*, metal and oxygen in the case of surface metal oxides) in the system, in addition to *p* and *T*. The most stable system geometry and composition is determined by the minimum surface free energy:
1

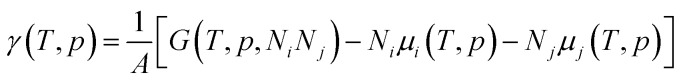

where *μ*_*i*_ and *μ*_*j*_ are the chemical potentials of individual components. It is assumed that there are separate reservoirs for each of the components. The surface free energy defined above represents the cost to form the particular surface structure (configuration) from the corresponding reservoirs. Finding the thermodynamically most stable configuration at given *T* and *p* is thus realized by finding the surface configuration that minimizes *γ*(*T*,*p*). Therefore, it is sufficient to calculate just the excess surface free energy with respect to a suitably chosen reference system:^152^
2

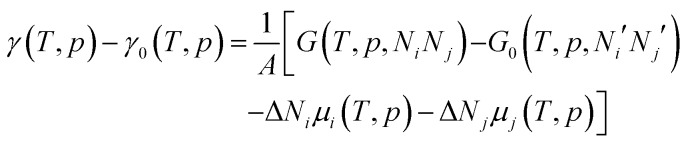

where *γ*_0_ and *G*_0_ are the surface free energy and Gibbs free energy of the reference system containing *N*_*i*_′ and *N*_*j*_′ atoms.

To avoid demanding calculations of Gibbs free energies it has been shown that it can be safely approximated by total DFT energies *E*^total^(*V*,*N*_*i*_,*N*_*j*_) calculated for volume *V*.^150^ First, the *pV*(*T*,*p*,*N*_*i*_,*N*_*j*_) term required for the transition from Gibbs to Helmholtz free energy was shown to be negligible (on the order of 10^–3^ meV Å^–2^). Second, the vibrational contribution to surface free energy *F*^vib^(*T*,*p*,*N*_M_,*N*_O_) was analyzed and the upper limit was estimated to be ±10 meV Å^–2^. Accepting these approximations makes the search for the thermodynamically stable surface computationally reduced, to the evaluation of energy difference:

3 Δ*E*^tot^ = *E*^tot^(*N*_*i*_,*N*_*j*_) – *E*tot0(*N*_*i*_′,*N*_*j*_′) – Δ*N*_*i*_*E*^tot^(*i*) – Δ*N*_*j*_*E*^tot^(*j*)

where individual terms on the right-hand side are DFT total energies calculated for the surface structure under investigation, reference system structure, and for reservoirs of components *i* and *j*. Taking surface metal oxides as an example, *E*^tot^(*i*) is the DFT energy of the O_2_ molecule in the gas phase and *E*^tot^(*j*) is the DFT energy of the bulk metal. All the temperature and pressure dependences in eqn (2) are contained in the remaining part of the excess surface energy Δ*N*_*i*_Δ*μ*_*i*_(*T*,*p*).

### 3.2. Structure of catalysts in a reaction environment

Within the approximations outlined above the constrained *ab initio* thermodynamics is computationally affordable and, thus, it becomes more and more popular in the catalysis community. The AITD approach has originally been introduced as a method for analyzing the chemical composition of the open catalyst surfaces under varying reaction conditions and it is currently routinely employed for predicting the most stable surface termination of complex multicomponent systems^155^–^158^ and for studying active site speciation in confined space of microporous crystalline materials.^159^–^162^

**Fig. 6:**
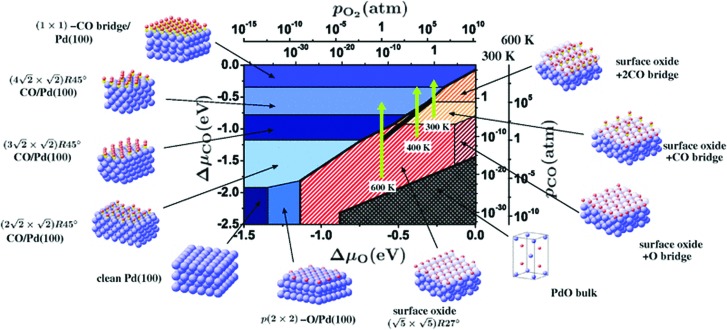
Surface phase diagram for the Pd(100) surface in “constrained thermodynamic equilibrium” with an environment consisting of O_2_ and CO. The atomic structures underlying the various stable (co)-adsorption phases on Pd(100) and the surface oxide are crosshatched, phases involving the surface oxide are hatched. The dependence on the chemical potentials of O_2_ and CO in the gasp phase is translated into pressure scales at 300 and 600 K. The thick black line marks gas phase conditions representative of technological CO oxidation catalysis, *i.e.* partial pressures of 1 atm and temperatures between 300–600 K. Reprinted with permission from ref. 154. Copyright 2007 American Physical Society.

An illustrative example for this method is a comprehensive study by Scheffler and co-workers on the composition of the Pd(100) model catalyst in a reactive environment corresponding to CO oxidation.^154^ The thermodynamic stability of various (relevant) surface structures formed under an O_2_ and CO atmosphere on the Pd(100) surface was investigated as a function of temperature and chemical potential of individual components. A combinatorially representative set of 119 ordered adsorption phases of O and CO on metal Pd(100) and oxide PdO(101)-
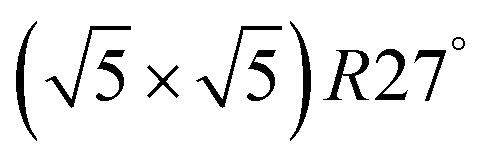
 surfaces were considered. Only 11 of them were found to be thermodynamically most stable structures for particular windows in (*T*, *μ*_CO_, *μ*_O_2__) space. The calculated surface phase diagram is depicted in Fig. 6. The bottom left corner corresponds to vanishing pressure of both CO and O_2_ gases and the clean Pd(100) surface is the most stable phase. Moving from left to right corresponds to increasing O_2_ pressure (and no increase of CO concentration). First, a p(2 × 2)-O/Pd(100) surface is formed where O atoms occupy the hollow sites on the Pd(100) surface with a corresponding oxygen coverage of *θ* = 0.25 ML. With increasing O_2_ pressure the 
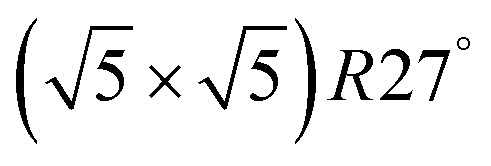
 surface is formed. At even higher oxygen concentration the PdO bulk becomes the most stable phase. Similarly moving from the bottom left corner upwards the thermodynamically stable ordered CO adsorption structures on Pd(100) are apparent. When both O_2_ and CO partial pressures increase there are three stable phases where O or CO is adsorbed on the 
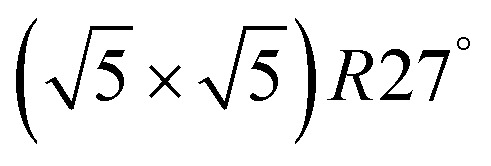
 surface oxide. However, no phase with both O and CO co-adsorbed on the 
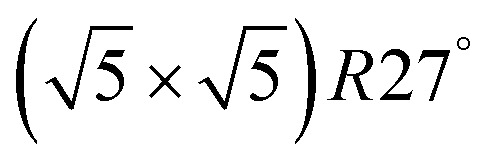
 surface was found (in agreement with experimental data^163^). Experimental conditions relevant to the technological CO oxidation catalysts were found to fall close to the boundary between phases derived from gas adsorption on 
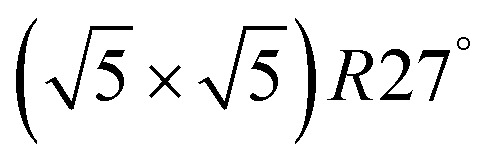
 surface oxide and from gas adsorption on the Pd(100) surface. Two important conclusions were drawn from this phase diagram: (i) thick bulk-like PdO oxide is not formed on the surface under technologically relevant conditions and (ii) the 
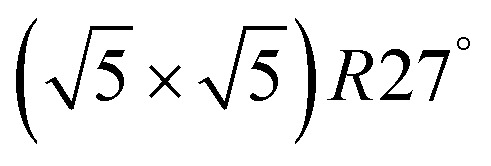
 surface oxide must be taken into consideration with respect to the catalytic activity of Pd under “*operando*” conditions.

The stability of the phase diagram with respect to changed exchange–correlation functional was also investigated.^154^ Comparing the results obtained at the LDA, PBE and rPBE levels, the topology of the phase diagram remained unchanged. However, individual phase boundaries are shifted one way or the other, depending on the functional employed. The differences were partly tracked down to the description of the gas-phase O_2_ and CO molecules. The constrained *ab initio* thermodynamics results reported above helped in identifying the relevant phases that must be considered for the assessment of the catalytic activity of the catalyst. They were subsequently used in first principles kinetic Monte Carlo simulations. Results of this study helped in understanding the relevant experimental data and they clearly showed that the catalyst surface (active state) can be dramatically changed moving from the UHV region to the “*operando*” conditions.

**Fig. 7:**
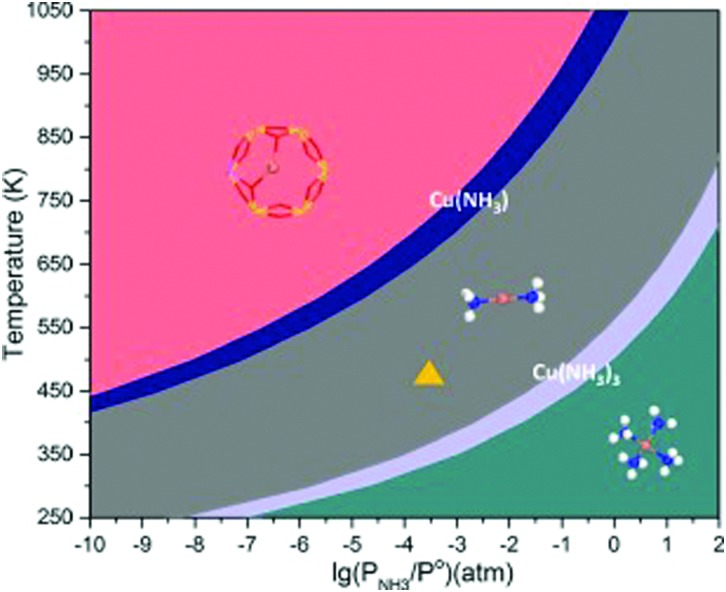
Phase diagram for Cu(NH_3_)_*x*+_ in CHA with varying NH_3_ pressure and temperature. The yellow triangle indicates typical operating conditions, *i.e.* a temperature of 473 K and an NH_3_ concentration of 300 ppm. The phase-diagram is constructed with NH_3_ in the gas-phase as reference. Reprinted with permission from ref. 164. Copyright 2018 The Royal Society of Chemistry.

As a second example we discuss a relatively simple but straightforward application of *ab initio* thermodynamics on a NH_3_-SCR process catalyzed by a Cu-CHA catalyst: phase of Cu^I^ under the reaction conditions.^164^ The catalytic reduction of NO_*x*_ is an environmentally important process increasingly enforced by legislation. The current approach for NO_*x*_ to N_2_ conversion is the selective catalytic reduction with NH_3_ as the reducing agent (SCR-NH_3_). Chen *et al.* used a combination of *ab initio* thermodynamics and *ab initio* molecular dynamics to elucidate the structure of the active species and mechanism of O_2_ activation in the Cu-CHA zeolite catalyst (see Fig. 7). The key question addressed by *ab initio* thermodynamics was the location and coordination of Cu^I^ ions in CHA under the reaction conditions (temperature and NH_3_ partial pressure). Previous experimental studies indicated that at temperatures below 523 K and above 623 K the SCR-NH_3_ activity showed second and first order dependence on the Cu loading.^165^

The state of the copper ions under SCR conditions was investigated by a thermodynamic analysis, constructing the phase diagram of Cu^I^ coordinated to increasing number of NH_3_ molecules. Following the strategy of Reuter and Scheffler^150^ the free energy difference between solvated and framework-coordinated Cu^I^ ions was obtained from:

4 Δ*G* = *G*_Cu(NH_3_)_*x*__ – *G*_Cu_ – *xμ*_NH_3__(*T*,*p*)

where *G*_Cu(NH_3_)_*x*__ and *G*_Cu_ were free energies of Cu^I^ ion solvated by *x* molecules of NH_3_ and Cu^I^ ions coordinated to the zeolite framework, respectively, and *μ*_NH_3__ was the ammonium chemical potential. Free energies were calculated with explicitly included zero-point energy corrections and vibrational entropies, within the harmonic approximation.

The phase diagram shown in Fig. 7 shows that at low temperature and high ammonia partial pressure the Cu^I^ ions are solvated by 4 NH_3_ molecules while at high temperature and low ammonia partial pressure they are coordinated to the framework oxygen atoms in the vicinity of framework Al. Under the conditions typical for the SCR-NH_3_ process the Cu^I^ ions are linearly coordinated to just two NH_3_ molecules and these Cu^I^(NH_3_)_2_ species are catalytically active species in O_2_ activation. Chen *et al.* also considered the fact that Cu^I^(NH_3_)_*x*_ species formed inside the zeolite channels and in the external gas phase may have different stabilities. The phase diagram constructed with respect to such a fluid phase (shown in ESI of ref. 164) is shifted in favor of Cu^I^ extra-framework cations; however, the conclusions about the character of the catalytically active Cu^I^(NH_3_)_2_ species remained unchanged.

Various applications of *ab initio* constrained thermodynamics have appeared in the literature during the last decade. The structures and stabilities of various nanoparticles used in catalysis were investigated as a function of synthesis conditions or “*operando*” environment, *e.g.* Mo_2_C, ZnO and Ni_2_P,^166^–^169^ surface coverage as a function of *T* and *p*_*i*_,^161^,^170^–^172^ doping effects,^173^ metal alloying,^174^,^175^ surfaces under electrochemical conditions,^176^,^177^ water in the interlayer space,^178^ or controlled growth conditions,^179^ to name just some. Several recent reviews and perspectives are also available.^152^,^153^,^180^–^184^

In summary, the *ab initio* constrained thermodynamics approach in the modeling of heterogeneous catalysts is a clear success of present-day computational investigation of catalysis. It brings a major step forward from 0 K/UHV conditions towards much needed “*operando*” modeling. As discussed in the introduction, such a step is inherently connected with increased complexity in modeling. The predictive power of *ab initio* constrained thermodynamics depends on the selection of particular surface configurations explicitly considered in the investigation. The use of global optimization techniques (described in the previous section) for the configuration selection with the *ab initio* thermodynamics is a major step towards minimizing the risk connected with missing important configurations. The accuracy of *ab initio* thermodynamics depends on the accuracy of the approximations involved. The choice of exchange–correlation functional is important for quantitative predictions; however, it has been shown that the qualitative picture remains unchanged regardless of the exchange correlation functional (see above).^154^ The approximation of Gibbs free energy by DFT total energies discussed above has been shown to be adequate (*e.g.*, ref. 150 or 168), giving an error of a similar or smaller size as the precision of the underlying exchange–correlation functionals. However, this assumption may not necessarily hold for all systems.

## 4. Free energy techniques

The transition from the potential-energy surface (PES) to the free-energy surface (FES) is essential for understanding catalysis (or chemical reactivity in general). Two classes of methods allowing a transition from PES to FES are discussed below. A conceptually and computationally (relatively) simple approach based on calculations of the Hessian matrix is covered briefly in Section 4.1 while computationally much more demanding biased molecular dynamics techniques are discussed in detail in Section 4.2.

### 4.1. Hessian-based thermal corrections

The transition from PES to FES can be simplified by construction of approximate partition functions for relevant stationary points on the PES. Since this is a well-established technique that has been used in computational chemistry (and catalysis) for decades we will only briefly outline its advantages and limitations.

Within the Hessian-based thermal corrections approach, partition functions are constructed for stationary points. The ideal gas expressions are typically used for translational and rotational contributions of free molecules while the harmonic approximation is used for vibrational partition functions. Thus, the evaluation of the matrix of second derivatives of energy with respect to atomic coordinates becomes the computationally most demanding part within this approximation. Since such calculations are expensive for systems with hundreds or more atoms, suitable approximations have been established. Partial Hessian vibrational analysis^185^ can greatly reduce the computational requirements with only a small error in calculated characteristics with respect to reference full Hessian vibrational analysis.

Gibbs or Helmholtz free energies can be obtained from partition functions in principle for any temperature; however, it should be noted that the validity of such extrapolation decreases with increasing temperature. An obvious problem is in the harmonic approximation used in the construction of vibrational partition function, in particular, for low energy modes. It has been recently shown that partition functions based on anharmonic vibrational frequencies lead to improved rate constants and other characteristics of catalytic systems.^186^–^188^ However, evaluation of vibrational frequencies beyond the harmonic approximation is associated with significant computational expense. A problem of low energy modes can be partially overcome with the mobile adsorbate method within which the low energy vibrational mode is treated as translational or rotational degree of freedom.^189^

The Hessian-based thermal corrections model allows accounting for realistic temperature, leaving the system composition and complexity unchanged; it is thus placed vertically above the 0 K/UHV model in Fig. 1. It follows that this method is suitable for the description of systems where (i) the structure of the catalyst active sites does not depend on the temperature and reaction environment, (ii) concentration of reactants is low (gaseous reactions) and (iii) reaction temperature is moderate. An important class of catalysts where Hessian-based thermal corrections have been successfully employed are zeolites; they are thermally and chemically stable in the catalytic systems in which they are used, and due to their microporous character the concentration of reactants at the active sites is limited. For more details see the recent review by Van Speybroeck *et al.*^190^

### 4.2. Biased molecular dynamics

With increasing temperature and complexity of reacting systems (*e.g.*, reactions at the liquid/solid interface), one needs to switch from descriptions based on a few individual configurations to a statistical description over ensembles representing reactant, product and transition state configurations. In other words, one moves from the potential energy to the free energy surface, which is the true landscape to be investigated. The free energy *F* is defined

5 *F* = –*k*_B_*T* ln *Z*

6

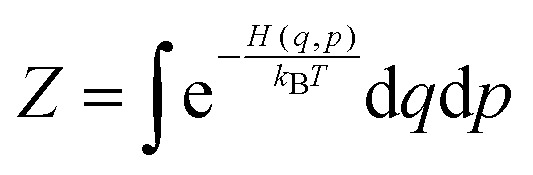

where *Z* is the partition function, *k*_B_ is the Boltzmann constant and *H*(*q*,*p*) is the Hamiltonian of the system. However, absolute free energies are typically not calculated since, with the exception of very simple systems, accurate evaluation of the phase space integration in eqn (5) is infeasible. Rather, the free-energy differences between (macro)states (reactant state, product state, transition state – separatrix, *etc.*) are calculated. Various approaches to sample representative ensemble of configurations and to accurately estimate free energy differences have been devised and are discussed in detail below.

The temperature-dependent configuration ensembles can be generated by molecular dynamics (MD) or Monte Carlo methods.^191^ However, within the context of first-principles description of reactive systems, Monte Carlo methods were used very little^192^,^193^ due to their typically low efficiency (low acceptance rates, highly correlated data series) and inherent inability to provide temporal characteristics (mechanism, kinetics). Therefore, *ab initio* molecular dynamics (AIMD) is nowadays a standard tool to study reactions on the free energy surface. Using plain AIMD for this purpose is, however, problematic, since most of the chemical reactions occur on very long time scales compared to elementary molecular motions which need to be described with AIMD. Thus, statistically significant sampling of these rare events in a plain AIMD is, with current computational resources and technology, largely intractable. To overcome this disparity in time scales and enhance sampling of these highly activated regions, a number of methods have been proposed and we refer the reader to topical reviews^194^–^196^ for an in-depth discussion. Here, the focus will be on two classes of enhanced AIMD methods which have been used extensively to obtain free energy reaction profiles within the field of catalyzed heterogeneous reactions: (i) methods using a biasing potential such as umbrella sampling^197^ or metadynamics,^198^ and (ii) thermodynamic integration.^199^

The metadynamics (MTD) is the most popular choice in catalytic applications not only from the first class of methods but also in general.^155^,^200^–^231^ The most relevant MTD applications will be discussed below. In MTD, an adaptive biasing potential is added on-the-fly during the simulation. The biasing potential is gradually accumulated from small repulsive Gaussian-shaped hills. The Gaussian hill width, height and frequency of deposition are, in the original formulation,^198^ fixed during the simulation and act as free parameters that need to be tested for the system at hand. The free energy profile can be estimated directly from the negative of the biasing potential. Numerous modifications of the original MTD have been developed^232^ since. In well-tempered MTD,^233^ the bias deposition rate automatically decreases with time, leaving the user with a single free parameter to test. In addition, the well-tempered formulation diminishes both the problem of when to stop the simulation and the problem of “hill surfing”, *i.e.* the behavior whereby the biasing potential overfills the underlying free energy surfaces and pushes the system into high-energy regions. This modification has already found application within the heterogeneous catalysis field in the study by Ghoussoub *et al.*^231^ on CO_2_ reduction *via* surface frustrated Lewis pairs of hydroxylated indium oxide. An important technical improvement of MTD is the multiple walkers implementation^234^ which enables running a number of MTD simulations in parallel on the same free energy surface, which all contribute to the overall history-dependent biasing potential. This technique ported to a supercomputer infrastructure has been successfully used to unravel the highly complex network of reaction pathways leading to the synthesis of methanol on ZnO^206^,^211^ and Cu/ZnO^222^ catalysts, accumulating about 2 ns of AIMD simulation time. Lately, a metadynamics-based collective variable-driven hyperdynamics^235^ has been employed to study plasma-induced surface charging effects on CO_2_ activation on supported M/Al_2_O_3_ (M = Ti, Ni, Cu) single atom catalysts.^236^ Importantly, most of the MTD-type schemes proposed are implemented and made easily available *via* PLUMED,^237^ an external plugin that can be interfaced with MD codes through a simple patching procedure.

Besides MTD, other enhanced AIMD schemes that use a biasing potential, including umbrella sampling^197^ or integrated tempering sampling (ITS),^238^ have also been employed in the field,^239^–^244^ albeit only scarcely. In a typical umbrella sampling simulation, a set of fixed biasing potentials is introduced spanning the entire region of interest in the order parameter. A common choice of potentials is a set of uniformly distributed harmonic functions with some overlap. For each overlapping region, or ‘window’, an AIMD simulation is performed yielding a set of partially overlapping histograms for the biased system. The unbiased free energy profile is recovered from biased histograms using schemes such as the weighted histogram analysis method^245^ or the dynamic histogram analysis method.^246^ The most notable examples of umbrella sampling applications are the QM/MM study on benzene hydrogenation on molybdenum carbide nanoparticles in benzene solvent^242^ and Car–Parrinello molecular dynamics (CPMD) based determination of free energies of methanol and water dissociation over TiO_2_ surfaces.^240^ Lastly, in ITS simulations the particles move in the effective potential corresponding to generalized distribution composed of a weighted sum of normal Boltzmann distributions at a series of temperatures around the target temperature. The free energy profiles can be extracted from ITS simulations by a proper re-weighting scheme.^247^ This approach, thus, mimics the replica-exchange^248^ type of simulations, in which the enhanced sampling is achieved by running a number of parallel trajectories at different temperatures with a Metropolis-type criterion for exchanging configurations. The appealing property of ITS, unlike other methods such as MTD or umbrella sampling, is the fact that it enhances sampling of all degrees of freedom, doing away with the non-trivial task of choosing a representative order parameter (see below), or so-called collective variable (CV). Combination of replica-exchange with MTD has also been proposed.^249^ However, the downside is the limited height of barriers that can be crossed using temperature as a switching parameter. Nevertheless, it was shown to be a suitable approach for low-energy activated processes such as carbene decomposition on a Ni(111) surface^244^ or CO diffusion on a Ru(0001) surface.^243^

Thermodynamic integration (TI) has also been extensively used for description of catalytically relevant systems,^250^–^265^ although not as much as MTD. It relies on calculating and subsequently integrating the derivatives of free energy with respect to a reaction coordinate along a reaction path. It can be shown that free energy derivatives are equal to a restorative force acting on the reaction coordinate, hence the alternative name for TI – Potential of Mean Force method. In practice, the integral is typically approximated by a quadrature with quadrature points regularly spaced along the reaction path. The free-energy derivative at each quadrature point is obtained from a constrained AIMD simulation, the blue-moon ensemble method,^266^ with the reaction coordinate value fixed.

Choosing between MTD-type methods and TI is not a simple task, as both have their weaknesses and strengths. TI is free of tunable parameters, with the possibility of systematically decreasing the statistical errors along the whole reaction path just by adding more sampling points and/or prolonging the simulation time. In addition, extraction of both kinetic and entropic information is rather straightforward^264^ in TI, which is not the case for MTD.^232^ However, MTD is much better suited to explore higher dimensional free energy surfaces characterized by multiple collective variables (typically only 2-D or maximally 3-D surfaces^206^,^222^ are explored due to increasing computational costs). As a result, the MTD and TI were sometimes^155^,^208^,^212^,^217^,^225^,^258^,^259^ used side by side with TI applied for simple reactions well-described by a single CV, while MTD was employed for more complex ones better described by two CVs.

**Fig. 8:**
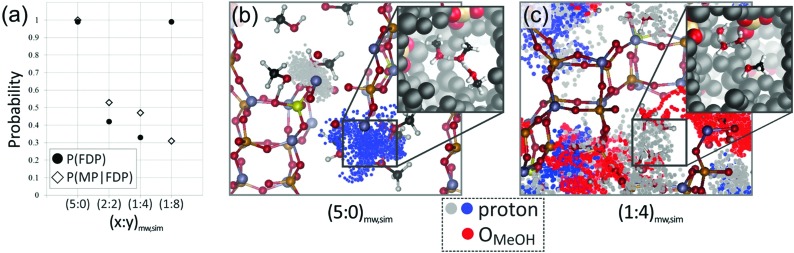
(a) Probability for framework deprotonation (FDP) and probability of methanol protonation when the framework is deprotonated (MP|FDP) during 50 ps MD simulations of different methanol–water mixtures adsorbed in H-SAPO-34 at 330 °C and around ambient pressure. (b and c) H-SAPO-34 loaded with a (5 : 0)mw,sim and (1 : 4)mw,sim methanol–water mixture. The gray, blue, and red dots represent the positions of the two acid protons and methanol oxygen atoms, respectively. The insets show snapshots of the MD run with highlighted acid sites. (*x*:*y*)mw,sim stands for a simulation with *x*MeOH and *y*H_2_O molecules per Brønsted acid site. Reprinted with permission from ref. 228. Copyright 2016 American Chemical Society.

Notwithstanding the exact dimension of still a very low-dimensional CV space that can be sampled in the enhanced AIMD schemes discussed so far, a good choice of the CV (or a small set of CVs) is essential for obtaining meaningful insight into the reaction. However, a choice of CV properly describing the true reaction process is not simple^267^ and no general purpose formula exists to obtain it. The problem is exacerbated for reactions, in which (i) solvent degrees of freedom are expected to play a role in the reaction mechanism, and (ii) a number of competing mechanisms are envisioned without a clear *a priori* preference for a specific one. One of the most often used solutions for both problems is to employ the atomic coordination numbers (CN) as the CVs, which are often flexible enough to accommodate various reaction scenarios. For example, using the CN of the carbon atom in methanol with oxygens of the surrounding water or methanol molecules as CVs, De Wispelaere *et al.*^228^ studied the role of solvent in the methanol-to-olefin process over a H-SAPO-34 microporous material (see Fig. 8). Similarly, Martínez-Suárez *et al.*^222^ employed three CNs (CN[C–O], CN[O–H], CN[C–H]), to investigate the complex reaction network of methanol synthesis over a Cu/ZnO nanocatalyst characterized by numerous competing mechanisms with a number of distinct C_1_ species identified. There are also a couple of methods to study catalytic reaction dynamics in an unbiased way, circumventing the problem of the correct choice of reaction coordinates, namely the quasiclassical trajectory (QCT) simulations^268^ and transition path sampling^269^ (TPS). In QCT, starting from the previously identified transition state a set of MD trajectories are propagated in an unbiased way, with the initial velocities chosen using quantum-mechanical population of vibrational states at a chosen temperature. Such simulations are particularly important for cases where the zero point vibrational energy (ZPVE) is large, since ZPVE is neglected in AIMD simulations where nuclei are treated as point charges moving on the electronic potential energy surface. Similarly, TPS creates an ensemble of unbiased reactive trajectories starting from the initial reactive trajectory, performing an important sampling in the trajectory space. The reactive trajectories corresponding to different reaction mechanisms are represented in the ensemble in proportion to the relative likelihood of the system to choose the particular mechanism, taking into account the effects of entropy and temperature. Both methods are capable of providing both the product selectivities (corresponding to differences in free energies of the products) and kinetic reaction rates for complex reaction networks. However, these methods also incur significant computational costs associated with a need to generate thousands of AIMD trajectories of a few ps in length to obtain converged results. Hence, they are mostly used as a tool for a qualitative understanding of complex reaction networks, possibly guiding another more quantitative investigation using, *e.g.*, enhanced sampling AIMD methods such as in the case of propane cracking over acidic chabazite by Bučko *et al.*^255^ The application of both methods in the heterogeneous catalysis field has focused so far exclusively on reactions in acidic zeolites, investigating linear hydrocarbon cracking in H-MFI^270^,^271^ and in H-CHA,^255^ alkane dehydrogenation^252^ and methanol coupling^272^ in H-CHA, and alkene methylation by methanol in H-MFI.^273^

#### 4.2.1. Physisorption and chemisorption

The catalytic process typically starts with the formation of a reactant/adsorption complex. With increasing temperature, the characterization of this complex using a single configuration becomes problematic. A need for statistical treatment is particularly important for molecules with rather weak and non-specific interactions with the catalyst as was first exemplified in the study of Bučko *et al.*^255^ The authors quantified the temperature effects on physisorption of propane in H-CHA zeolites using only equilibrium MD, which is sufficient for physisorbed systems. They showed that at low temperatures (100 K) the propane is bound to a Brønsted acid site but at 800 K, a typical catalytic cracking temperature, propane is mostly detached from the catalytic site with its movement in the zeolite being much less restricted. This temperature-dependent change in adsorption behavior is associated with a significant decrease of adsorption energy of about 20 kJ mol^–1^. The follow-up study by Göltl *et al.*,^274^ extended to other alkanes and employing higher levels of theory, confirmed the previous findings of Bučko *et al.* In addition, Göltl *et al.* proposed a simplified approach to obtain dynamically averaged adsorption energies from shorter equilibrium MD simulations. A cheaper way to include the temperature corrections to adsorption enthalpy and entropy was used by Tranca *et al.*^271^ who complemented their static DFT calculation with temperature corrections derived from Monte Carlo simulations using empirical force fields. In line with experimental data and previous studies by Bučko and coworkers^255^,^274^ a decrease of adsorption enthalpies with increasing temperature and temperature dependence of adsorption entropies were reported. A similar approach to that of Tranca *et al.*^271^ has been recently used by Van der Mynsbrugge *et al.*^193^ to investigate the influence of pore geometry on monomolecular cracking and dehydrogenation of *n*-butane in various acidic zeolites. The effect of spatial constraints on the free energy of adsorption was also studied by Bučko *et al.*^257^ who probed propane adsorption in two pores of different dimensions in H-MOR zeolites. Their free-energy profiles from TI using the blue-moon ensemble clearly showed that with increasing temperature from 0 to 800 K the entropy shifts the balance towards propane occupying the less confined pore, despite the larger adsorption enthalpy in the smaller pore.

**Fig. 9:**
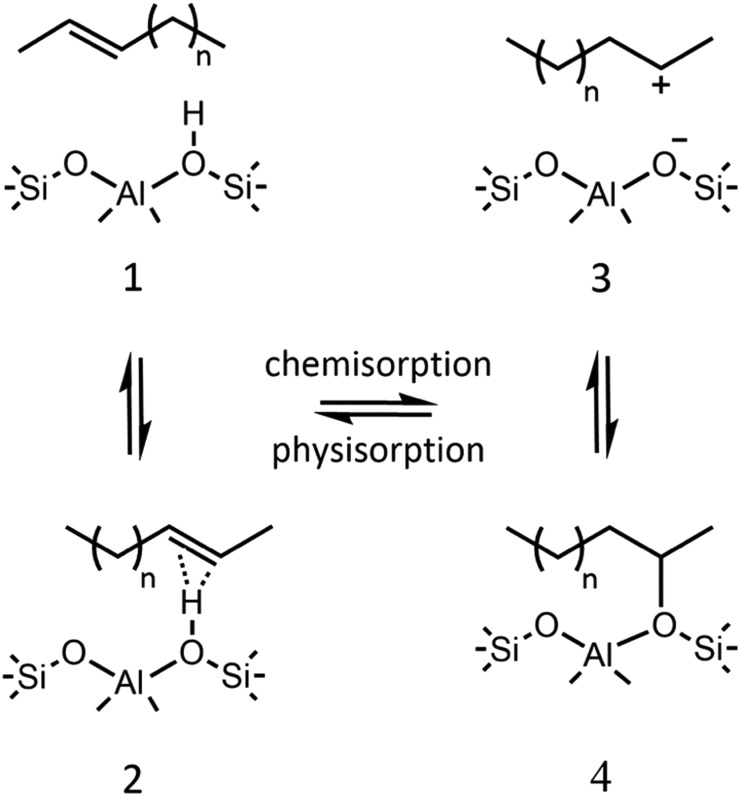
Illustration of the different intermediates upon alkene (2-pentene) adsorption in the presence of a Brønsted acid site (BAS): (1) physisorbed van der Waals complex, (2) alkane π-complex, (3) chemisorbed carbenium ion and (4) chemisorbed alkoxide. Reprinted with permission from ref. 230. Copyright 2017 Elsevier.

Moving beyond physisorbed complexes but staying in the zeolite hydrocracking field, Hajek *et al.*^229^ showed how inclusion of temperature effects beyond the harmonic approximation changes the relative stabilities of four types of pentene adsorption complexes in acid zeolite H-ZSM-5 including both physisorbed and chemisorbed species. Using only the harmonic approximation, the so-called π-complex, a complex bound to Brønsted acid sites *via* a C=C double bond, is found to be the most stable species at 323 K (see Fig. 9). However, upon inclusion of dynamical effects from equilibrium MD, stability of chemisorbed species, an alkoxide, becomes basically equal to that of the π-complex. The MTD-based free energy profiles for the π-complex → alkoxide transformation confirmed this finding, showing also that formation of the chemisorbed complex is an activated process with a barrier of approximately 40 kJ mol^–1^. In the following year, the same group^230^ extended their (biased) AIMD investigation to other C_4_–C_5_ alkenes focusing on dynamical effects at operating temperatures of catalytic alkene cracking of about 800 K. They found that another chemisorbed species, an ion-pair called the carbenium ion, is the prevalent species in the zeolite channels under these conditions, in stark contrast to predictions from static calculations favoring the π-complex. The change in stabilities of various species was attributed to entropy effects which may disfavor the formation of tightly bound physisorbed or chemisorbed complexes such as the π-complex or alkoxide. This is in line with the observation that the stabilization of the carbenium ion relative to other species increased from primary to branched C_4_–C_5_ alkenes. Recently, a general approach for estimation of adsorption free energies in zeolites based on enhanced AIMD simulations using TI has been proposed^263^ and applied for adsorption of small-molecules at Cu sites in chabazite.

Another aspect of a catalytic process, co-adsorption of reacting species, was studied by De Wispelaere *et al.*^228^ providing insight into water–methanol and water–propene competition for catalytic sites in a H-SAPO-34 microporous material, an important catalytic system for the methanol-to-olefin (MTO) process. Despite larger adsorption enthalpies of methanol than water, obtained both from static and dynamic calculations at 330 °C, the equilibrium MD runs with methanol–water mixtures showed that water and methanol have nearly equal probability to occupy the Brønsted acid site. As a result, the mixed methanol–water clusters compete for the acidic proton and exhibit lower apparent proton affinity than either of the pure systems (see Fig. 8). This means that methanol protonation, an elementary activation step for the MTO process, is slowed down in water. Similarly, the water is reported to displace propene from Brønsted acid sites in the co-adsorption scenario, decreasing the probability that propene becomes activated for further reaction toward the formation of cyclic hydrocarbon pool species.

#### 4.2.2. Quantifying entropy effects in simple reactions

To understand the role of entropy and to assess the limitations of harmonic transition state theory (HTST) in heterogeneous catalysis it is instructive first to look at a series of simple reactions of hydrocarbons in acid zeolites studied by Bučko and coworkers.^251^–^253^,^257^ In their first study,^251^ Bučko *et al.* tried to understand the origin of experimentally observed regioselectivity in proton exchange of isobutane in acid zeolites, *i.e.* why the methine (CH) group in contrast to the methyl group of isobutane is completely inactive. While the activation free energies from HTST at 800 K were basically the same for both groups, the barriers from TI-based AIMD differed by 55 kJ mol^–1^ in favor of proton exchange *via* the methyl group. This effect was shown to originate in the entropic contributions and was clearly related to different steric restrictions for proton access to two types of carbon groups in the early stages of the proton transfer. The differences in entropy contributions correlated well with relative probabilities of methine and methyl group to form adsorption complexes with Brønsted acid sites, which could be obtained already from the unbiased molecular dynamics of the reactant state. Hence, the failure of HTST to account for the regioselectivity can be again (see Section 4.2.1) traced back to inadequate representation of the reactant state. In 2010, Bučko *et al.*^253^ briefly analyzed entropy effects for propane cracking both using TI-based AIMD simulations and the static harmonic approximation. The discrepancy between the two approaches for entropy estimation amounted to as much as 80 kJ mol^–1^ at 800 K. Moreover, the activation entropies from the two approaches differed even qualitatively, with HTST associating larger entropy with the transition state, a pentavalent carbonium cation, while AIMD showing that the entropy of the reactant state, a loosely bound propane in the cavity, is larger than that of the transition state. Again, the shortcomings of a static approach were related to an improper description of the loosely bound reactant state. Another shortcoming of the static approach, inability to account for reaction intermediates which are not potential energy stationary points, has been highlighted in the case of propane dehydrogenation in H-CHA zeolites.^252^ The TI-based AIMD study complemented by TPS simulations revealed a more complex mechanism than expected based on static TS search, which originated in entropic stabilization of the propyl cation, a non-stationary point on the potential energy surface. TPS simulations showed that the propyl cation is an important branching point in the dehydrogenation mechanism, undergoing various transformations (internal rearrangement, rotations, translation in the cavity) during its lifetime, eventually collapsing directly to various stable products such as propene, the main experimentally observed product. As a result, creation of the main HTST-based intermediate, the alkoxide, can be, and in most dynamical trajectories is, avoided. Lastly, the role of spatial constraints on the reactivity of propane in the zeolite catalyzed cracking was investigated^257^ in the model system of acid mordenite containing larger and smaller cavities. The activation free energies, derived from TI-based AIMD simulation, were lower for cracking in stronger confinement in the smaller cavity mostly due to different entropies of activation. In both cavities, the entropies of the TS ensemble are lower than those of the reactant ensemble, however, the reactant in the narrower pore is rather confined to start with, so the relative entropy loss is smaller for a narrower pore. This explanation was justified by the significantly higher collision probability between propane and the active site in the smaller pore. However, the reactant can access the small pore only from the larger one, which is an activated process that tilts the balance toward cracking in the larger pore, in line with the experimental observations. This model study nicely illustrates the intricacies of even rather simple reactions in confined environments and a complex interplay of entropic and enthalpic effects that need to be considered under the working conditions of a catalytic process.

The entropy effects and HTST limitations in simple reactions were analyzed and discussed also outside the field of zeolite catalyzed hydrocarbon conversions, albeit only to a limited extent. Sun *et al.*^244^ compared reaction entropies, free energies and reaction rates for carbene (CH_2_) decomposition on an Ni(111) surface obtained from harmonic approximation and enhanced AIMD simulations using integrated tempering sampling (ITS). The HTST values were qualitatively consistent with the results from ITS-AIMD simulations; however, rates were about an order of magnitude larger for HTST and HTST reaction free energies were about 0.15 eV larger. The authors proposed *ad hoc* generalization of HTST including multiple configurations in the partition functions of the reactant and product states, which improved the agreement with ITS-AIMD reaction free energies and entropies. In addition, Sun *et al.* tested some of the general assumptions underlying the generalized TST (non-recrossing, quasi-equilibrium between reactant and transition states) by comparing generalized TST rates obtained from biased ITS simulations and true reaction rates from a TPS-inspired unbiased approach,^275^ which directly samples the (un)reactive trajectories. Rates from both approaches were in very good agreement. Hence, their general conclusion was that, for such a simple surface reaction, the basic assumptions of generalized TST theory are valid but the harmonic approximation is an over-simplification even in this simple case. The importance of entropic effects was further highlighted in the umbrella sampling CPMD simulation of methanol and water dissociation on TiO_2_ surfaces.^240^ The authors reported that with increasing temperature, the dissociation of water on the anatase surface becomes more favorable than that of methanol due to entropic effects. The large entropy loss on the side of methanol was associated with hindered rotation of the methyl group after dissociation. As a last example, a conceptually nice case study of temperature effects was presented by Schnur *et al.*^241^ for H_2_ dissociation on water-covered Pt(111), Ru(0001) and Pd/Au(111) surfaces. They reported how distortions of initially ice-like hexagonal water structure over metal surfaces at room temperature lead to an increase in the free energy barrier for H_2_ dissociation by 0.15 eV. The increase in free-energy barrier is related to an irregular shape of the hexagonal water rings under thermal conditions which makes the propagation of the spherical H_2_ molecule through the water layer much harder.

#### 4.2.3. Complex reaction mechanisms and reaction networks

Many of the mechanistic studies using AIMD simulations focused on rather simple reactions, aiming primarily at properly quantifying the temperature effects for well-known reaction mechanisms, often with an assumption of a unique reactant/TS/product sequence. However, in many industrially relevant heterogeneous catalytic processes, complex reaction mechanisms with multiple reaction channels and side reactions are at play.

The first step on the way to simulate more realistic reaction processes is to allow for multiple transition states connecting the reactant/product pair. One can either: (i) consider more reaction channels chosen based on previous reports and/or chemical intuition, tailor mechanism-specific collective variables, evaluate free-energy profiles and compare, such as in the case of single atom catalysis of O_2_ activation and CO oxidation over Rh_1_/γ-Al_2_O_3_,^213^ or (ii) use a more bias-free approach such as TPS simulations to create unbiased reactive pathways which connect the reactant and product basins without a need to constrain the transformation mechanism search using collective variables. The latter approach has been used by Bučko *et al.*^255^ to choose between possible realizations of the first reaction step of protolytic cracking of propane using acid chabazite as a catalyst at realistic reaction temperature (*T* = 800 K). The free-energy profile of the dominant mechanism determined in TPS simulations was later refined by TI-based AIMD.

The possibility of forming multiple products from a single transition state represents an additional layer of complexity in the realistic reaction mechanisms. An approach specifically constructed to meet the challenge is quasiclassical trajectory (QCT) simulations shooting a set of unbiased MD trajectories from the TS, followed by the analysis of the end products of the simulations, which provides an estimate of the product distributions at operating temperature. The QCT has been used in pioneering studies of Bell and coworkers on product selectivities for alkane cracking^270^ and alkene methylation by methanol^273^ over acid zeolite H-ZSM-5. In both studies, the authors reported qualitative discrepancy between product distributions obtained by static and dynamic reaction pathways obtained from QST suggesting that the high temperature pathways, *i.e.* the free-energy pathways, differ significantly from 0 K potential surfaces. Besides QST, the TPS simulations, with a judicious choice of the order parameter that accommodates multiple product scenarios, may also be used to estimate product selectivities as shown for the propane dehydrogenation mechanism in H-CHA zeolites^252^ (see Section 4.2.2 for a more detailed discussion).

**Fig. 10:**
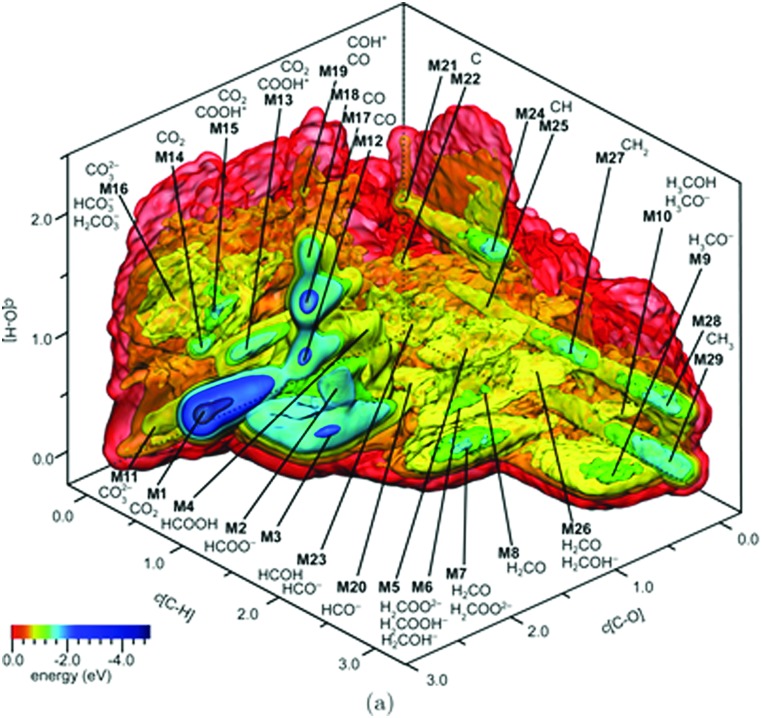
Free energy landscape from the metadynamics sampling of methanol synthesis based on CO_2_ over the reduced Cu_8_/ZnO(0001[combining macron]) catalyst surface model (a). The coordination numbers c[C–O], c[C–H], and c[O–H] (see text) were employed as collective variables (CVs) to describe the interaction of the carbon atom with the oxygen atoms of the top layer of ZnO and the two oxygen atoms of the reactant CO_2_, the carbon atom with all hydrogen atoms in the system, and all hydrogen atoms with the two oxygen atoms of the reactant CO_2_, respectively. Relative free energies Δ*F* are reported according to the shown color scale. Bold capital M plus a number labels distinct free energy minima. Reprinted with permission from ref. 222. Copyright 2015 American Chemical Society.

The first truly complex reaction network investigated using dynamical *ab initio* methods was the methanol formation from CO on a defective hydroxylated ZnO(0001[combining macron]) surface.^206^ The reaction network was investigated in a two-stage procedure starting with a multiple-walker metadynamics simulation with constraints on carbon diffusion into or away from the surface and a coarser biasing setup to speed up the exploration of the vast free surface. The gross free energy surface obtained from the exploratory mapping already contained the molecular species considered in previous studies and also yielded additional subspecies. In the second stage, the most important transformations were refined by individual one- to three-dimensional metadynamics runs with collective variables tailored for a particular transformation. Altogether, about ten stable intermediate species were identified being interconnected *via* five distinct reaction channels leading eventually to full hydrogenation of the CO molecule. In the follow-up study by the same group,^222^ the complexity of the catalytic system as well as its relevance for experiment was increased further by switching the focus to methanol synthesis from CO_2_ over a Cu/ZnO nanocatalyst, which was modelled using a Cu_8_ cluster deposited on an O-terminated and partially hydroxylated ZnO(0001[combining macron]) surface. The relevance of this catalyst model under conditions of industrial methanol synthesis was established from the surface phase diagram constructed using *ab initio* thermodynamics^276^ (see Section 3). Rather than providing an accurate free energy surface with converged barriers and reaction energies, the authors aimed at exploring the breadth of the reaction network identifying all possible types of C_1_ species (more than 20) and reaction channels present over the Cu_8_/ZnO(0001[combining macron]) catalyst (see Fig. 10). Their almost 2 ns long exploratory CPMD-based metadynamics run in three-dimensional collective variable space also included several well-known side reactions, such as coking, methanation and water-gas shift reactions. In addition, the study highlighted the need to systematically include the surface region at the interface of the catalyst and gas phase as an active reaction space since (i) the Cu cluster is highly dynamic at 500 K, changing from 2D planar structures lying flat on the ZnO surface to “spherical” 3D morphologies, with Cu atoms migrating across the catalytic system, (ii) there are strong-metal support interactions manifested in spontaneous creation of O vacancies, which migrate from atop the ZnO(0001[combining macron]) surface layer onto the Cu_8_ cluster, thus giving rise to O adatoms or to OH adspecies after a subsequent reaction of these O atoms with H adspecies from Cu_8_, and (iii) Cu atoms interact strongly with C_1_ species which may cause a spatial redistribution of some of these Cu atoms over the support. All these facts give rise to various active morphologies and new putative active sites created *in situ* that can stabilize reactant, intermediate, and product states of the involved C_1_ species. Admittedly, the degrees of freedom responsible for these “surface-reconstruction” processes are not accelerated by the metadynamics but occur on the time scale that is accessible only to nonbiased AIMD dynamics, which limits the configurational space of the catalyst transformations that could be accessed. Also, the AIMD setup did not allow for an on-the-fly insertion (or removal) of reactants in the sense of grand canonical equilibrium with suitable reservoirs for these molecules, which might be needed for faithful and automated description of the industrial process. Nevertheless, along with the reactive global optimization approach discussed in Section 2.5.3, this work, which couples *ab initio* thermodynamics with extensive biased AIMD simulations to faithfully map a complex reactive network under working conditions, presents one of the most comprehensive examples of using *ab initio* simulations to study catalytic processes.

## 5. Simulating reaction kinetics for mechanistic analysis and catalyst optimization

### 5.1. Basic principles

Previous sections have illustrated the power of modern computational approaches for unraveling the nature of catalytic ensembles and studying individual chemical transformations under realistic *operando* conditions. Yet, most of the mechanistic studies of practical catalytic reactions and detailed analysis of complex reaction networks commonly encountered in heterogeneous catalysis is still limited to electronic structure calculations on the 0 K/UHV models. Such an approach has been proven over the last two decades to be extremely powerful in unraveling the fine mechanistic details of the chemical transformations underlying heterogeneously catalyzed reactions.

In practice, even the simplest of such processes are represented by complex networks of competing and parallel elementary reaction steps involving different sites. Quantum chemical calculations provide direct access to the rate constants for each of these steps. However, the resulting information has often only a limited value by itself as the means to provide direct guidance for the optimization and design of an improved catalytic process. The next step in this direction requires the reduction of the mechanistic complexity and a bridge between the microscopic insights into surface reactions and the macroscopic kinetics of the catalytic process. This step can be readily accomplished through microkinetic modeling, which is currently one of the most popular and powerful approaches to analyze reaction mechanisms and reaction kinetics in both experimental and computational catalysis.^277^–^280^ Microkinetic models can be directly used to identify which intermediates or specific reaction paths dominate the formation of a particular product, providing a practical tool to directly optimize process conditions and even to guide the *in silico* design of improved catalysts. The theory and applications of first principles kinetic modeling have been extensively discussed in a number of excellent recent reviews.^281^–^286^ In this section, we therefore limit ourselves to only briefly outlining the fundamental approximations made in such methods and highlighting their power by discussing selected relevant examples from recent literature.

In microkinetic modeling, rate constants of elementary reaction steps are used in the mean-field differential equations that describe the kinetics of the reaction.^287^ The output of such a simulation is production rates and surface concentrations. The mean-field approximation implies that the adsorbates are not correlated spatially and therefore their mutual interactions are neglected. Although the development of more advanced kinetic modeling approaches accounting for such correlation effects is an active field of research,^288^–^290^ the conceptual simplicity and ease of their practical implementation determine the success and widespread utilization of the mean-field phenomenological kinetic modeling approaches.

The formulation of a microkinetic model begins by expressing all the rates of elementary reaction steps in a catalytic reaction network *via*
7

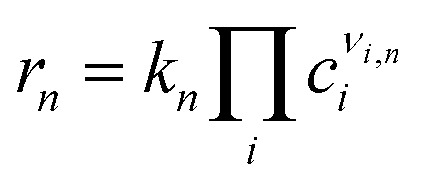

where *k*_*n*_ stands for the rate constant of the elementary reaction step *n*, *c*_*i*_ is the concentration of the component *i* and ν_*i*,*n*_ is the stoichiometric coefficient for species *i* in step *n*. The time-dependent concentration of the component or surface coverage *i* is calculated by
8

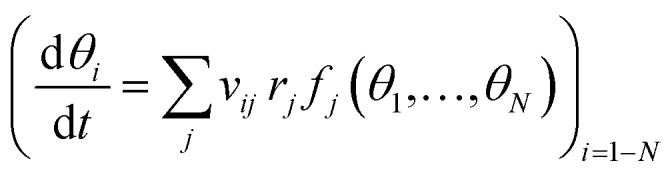

where *θ*_*i*_ is the surface coverage of species *i* at time *t*, *ν*_*ij*_ is the stoichiometric coefficient for species *i* in step *j*, *r*_*j*_ is the rate of the reaction *j* and *f*_*i*_ is a function of several coverages involved in step *j*. This system of differential equations describes effectively all chemical processes taking place in the catalytic system. In practice, one solves this system of equations numerically until a steady state is reached for the overall reaction system. In these equations, the rate constants are commonly computed in the framework of the transition state theory (TST) as
9

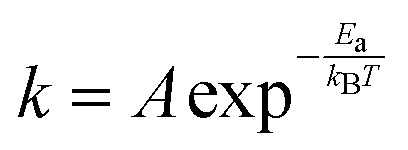

where *E*_a_ is the intrinsic activation barrier for a particular elementary reaction step readily accessible from DFT calculations, while the pre-exponential factor *A* can be represented as
10

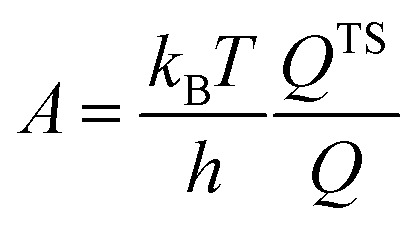

where *k*_B_, *h* and *T* are, respectively, the Boltzmann constant, Planck constant and the temperature, and *Q*^TS^ and *Q* are the partition functions of the transition state and reactant state, respectively.

These partition functions reflect the entropic effects associated with the chemical transformation and they can also be estimated from the results of DFT calculations by, for example, treating each degree of freedom in the reactive system by a frustrated vibration that is in turn treated as a harmonic function. Despite other more advanced approaches involving the explicit consideration of the translation degrees of freedom for the adsorbates and accurate sampling procedures allowing an increase in the computational accuracy manifold,^185^,^291^–^293^ semiempirical and phenomenological approaches for estimating pre-exponential factors provide useful practical solutions for constructing microkinetic models with a high predictive power.^294^–^298^

When the overall description of the catalytic system is constructed, the apparent activation energy (*E*appa) can be computed from the model as
11

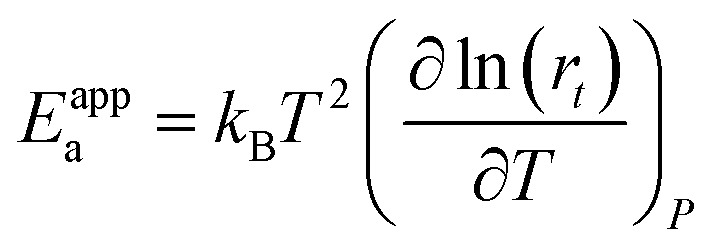

And the rate-determining step can be identified by using the degree of rate control (DRC) parameter^299^,^300^ that effectively describes how the overall kinetics is influenced by a particular elementary step *i* considered in a microkinetic model:
12

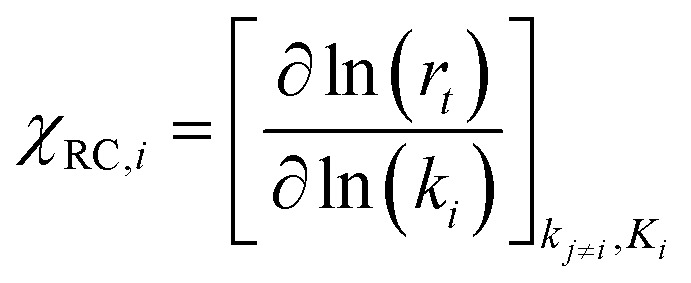




The DRC can be viewed as a weighing factor that allows directly relating such macroscopic rate parameters as the apparent activation energy and reaction orders and the microscopic characteristics as the elementary reaction rates and activation barriers for elementary steps.^27^ In principle, the DRC analysis method can be directly employed for studying and optimizing catalytic reactions. The important advantage of this methodology is that it does not require the complete derivation of the complete catalytic mechanism for its successful application.^302^

To summarize, microkinetic modelling is a powerful tool for analyzing complex reaction mechanisms and constructing predictive models capable of connecting the microscopic description of the reactive systems with measurable macroscopic activity descriptors. The mean-field approximation underlying these methodologies not only facilitates the analysis of the results, but also ensures the high efficiency of the associated calculations as well as its straightforward implementation in a working code. This has given rise to a number of programs for advanced microkinetic simulations and analysis of their results which are currently available to the scientific community.

### 5.2. Microkinetic modeling and linear energy relations for catalyst design

If the kinetic parameters for a large enough selection of catalyst candidates are available, microkinetic modeling becomes a practical computational tool for catalyst design and optimization. However, the explicit calculation of the activation barriers for different reactions and catalyst formulations using accurate electronic structure methods is a highly resource- and time-consuming task. The theory-guided catalyst design can be greatly assisted through so-called linear scaling relationships, which establish correlations between the adsorption energies of specific intermediates and the activation barriers for the related chemical transformations.^303^–^305^ The existence of such linear scaling relations has been demonstrated for a wide range of reactions over different catalysts.^306^–^308^ When such relations hold, they allow for a significant reduction of the number of independent parameters which determine the catalyst activity. They therefore facilitate enormously the *in silico* search for an optimal catalyst,^309^–^311^ but at the same time place fundamental limits on the maximum achievable activity or selectivity. The search for ways to break these scaling relations is currently an active research topic in computational catalysis.^301^,^312^,^313^

**Fig. 11:**
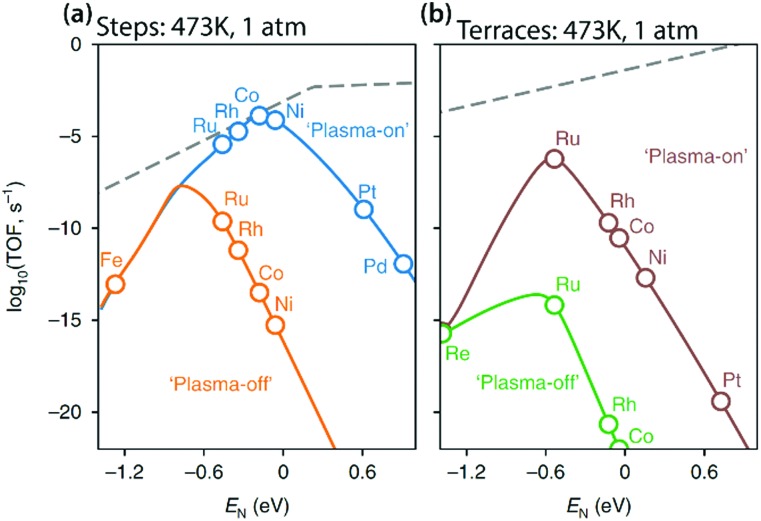
The calculated rates of ammonia synthesis on (a) step and (b) terrace sites under thermal (“Plasma-off”) and plasma-induced (“Plasma-on”) conditions. Reprinted with permission from ref. 301 Copyright 2018 Springer Nature.

As an illustrative example of the power of the integration of DFT modeling and microkinetic simulations for catalysis design, let us discuss one of the most classical and important catalytic processes – ammonia synthesis (N_2_ + H_2_ → NH_3_), where the catalyst performance is actually limited by such scaling laws.^303^ According to the Sabatier principle, an ideal catalyst for this process should be active enough to promote the cleavage of the strong bond in molecular N_2_ and, at the same time, bind various NH_*x*_ species rather weakly so that they can be removed from the surface by the hydrogenation as the NH_3_ product. However, because the adsorption energies for these intermediates and the activation energies for the elementary steps are correlated with each other, one cannot independently adjust them to maximize the performance. Microkinetic simulations based on the results of DFT simulations for a wide range of catalytic materials have revealed clear volcano-type relations between the catalytic performance and binding energy of atomic nitrogen (Fig. 11, “plasma-off”), which has been identified as a suitable reactivity descriptor for this process.^314^,^315^

In a recent work, Go, Hicks, Schneider and co-workers proposed a way to overcome the fundamental limitations imposed by such linear relations by coupling the conventional catalysis with non-thermal plasma.^301^ Indeed, the correlation between the adsorption and activation energies is directly related to the intrinsic chemistry of the catalytic materials. The thermocatalytic limit for ammonia synthesis was assessed through a microkinetic model based on the DFT-computed energetics for the ammonia synthesis reaction intermediates treated in the frozen-adsorbate limit and tabulated standard entropies for the gaseous reactants. This model was then adjusted to include the influence of the N_2_ vibrational excitation on the elementary reaction rates. It was proposed that the vibrational excitation of the gaseous N_2_ through the interaction with the non-thermal plasma would increase the energy of the initial state by the energy of vibration, resulting in an effective lowering of the associated transition state. This new model provided the initial evidence that the optimal catalysts and active sites in plasma catalysis may differ from those in thermal catalysis (Fig. 11 – “plasma-on”). Importantly, besides enhancing the overall rate of the catalytic reaction over the open terrace sites (Fig. 11b), the selective excitation of the N_2_ vibrational states of the reactant was shown to shift substantially the maximum of the volcano curve computed for the more reactive step-sites from the expensive Rh and Ru to the cheap and earth-abundant Ni and Co catalysts. These theoretical predictions were found to coincide very well with the experimentally determined reaction rates under plasma-induced catalysis conditions.^301^

### 5.3. Microkinetic modeling for deep mechanistic analysis and process optimization

The derivation of straightforward activity relations can be complicated by the high complexity of the chemical ensembles acting as the active sites. Effects such as substrate pre-activation and active site relaxation can induce deviations from the expected activity trends. Furthermore, processes such as the multiple-site activation, active site cooperativity and confinement-induced reactivity make the definition of simple reactivity descriptors suitable for the large-scale computational screening of different catalysts very difficult if not impossible.^317^

**Fig. 12:**
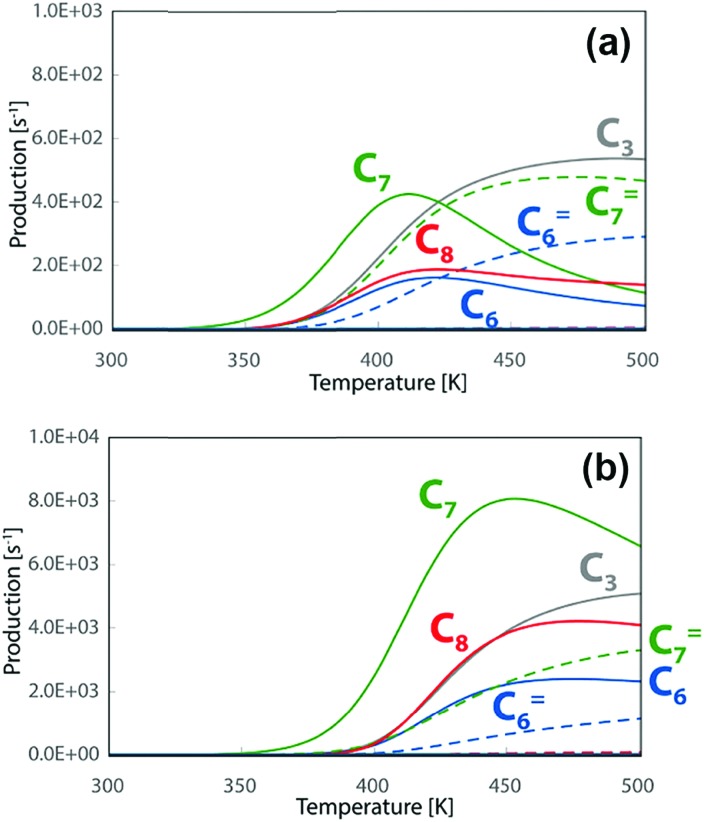
Microkinetics simulated production rates of the alkylation reaction by the La-FAU model at the total pressure of (a) 3.2 bar and (b) 32 bar as a function of temperature. The desirable reaction product is the C7 alkylate dominated by 2,2-dimethylpentane (which rapidly isomerizes to 2,3-dimethylpentane by a secondary reaction that was not included in the model) produced by the reaction of *tert*-butyl cation and propene stabilized inside the La-FAU pores. The second major C8 product is formed *via* the self-alkylation path. The formation of unsaturated hydrocarbons at elevated temperatures gives rise to oligomerization reactions inside the pores resulting in the pore blockage and rapid catalyst deactivation. Reprinted with permission from ref. 316. Copyright 2017, American Chemical Society.

In such cases, MKM can be used to reduce greatly the mechanistic complexity of the DFT-computed reaction networks and identify the optimal reaction conditions so that the desirable reaction path is enabled resulting in the enhanced selectivity of the overall catalytic process. A recent study by Liu *et al.* on the mechanism of isobutene–propane alkylation by faujasite-type zeolite catalysts illustrates such an approach.^316^ A detailed mechanistic DFT analysis of the extended catalytic network underlying the isobutene–propene alkylation process using realistic models of La-containing low-silica faujasite-type zeolite catalysts has been carried out. A particular focus was laid on enhancing the selectivity to the desirable alkylate product, while suppressing the paths which resulted in the deactivation of the zeolite catalysts. Microkinetic models were constructed, based on the DFT-computed energetics of the elementary reaction steps and augmented by configuration-bias Monte Carlo simulations to more accurately account for the relative concentrations of reactants at the reaction centers. The simulations clearly showed that the production of the desirable C7 alkylate over the La-FAU catalyst is favored when operating the reaction at a high pressure and low-temperature (Fig. 12). Furthermore, the mechanistic insights obtained through the MKM simulations pointed to the fundamental requirement of the micropore structure of the hypothetic optimal alkylation catalyst. Given the large size of the hydride transfer complex of isobutane and carbenium ions, a zeolite structure with large pore size should be beneficial. On the other hand, the microkinetic simulations highlighted the importance of high isobutane occupation in the zeolite micropores that would be optimally realized for the small-pore zeolites. Based on these conflicting requirements on pore size dimensions, the authors proposed that a bimodal channel structure should be looked for in an optimal catalyst.^316^

## 6. Boosting catalyst design with machine learning approaches

### 6.1. Machine learning in chemistry

Over the past few decades chemical science has produced gigantic amounts of data. This, aligned with the maturing of practical data science approaches and the enormous growth in computational power, has begun to render new Big Data strategies efficient for discovering new correlations, developing models, and making profound predictions.^318^ One of the most powerful strategies of this sort is machine learning (ML), the utilization of which for data analysis has spread rapidly in computational chemistry.^319^ The possibility of making fast predictions with accuracy comparable to conventional computational chemistry makes ML methods especially appealing, not only as a research tool but as a vital ingredient of the catalysis-by-design strategy. There is a noticeable expansion of ML approaches to data analysis in different areas of chemistry. ML models are currently employed for studying chemical reactions, predicting properties of different chemical substances, materials design and the development and testing of new continuous and discrete descriptors.^320^–^332^ Some of these approaches allow for the optimization of existing chemical reactions and even the discovery of yet unknown ones.^333^,^334^ Important applications of the ML techniques have also been witnessed in recent years in different branches of catalysis sciences.^305^,^329^,^335^–^338^

The availability of the vast, machine-readable and readily accessible scientific data, together with the enormous available computational power of modern CPUs and GPUs, which are capable of carrying out fast and cheap calculations, gives rise to a situation whereby ML approaches are becoming an important ingredient of rational design strategies for catalysis.^339^,^340^ So far, ML techniques have already been employed as an enabling technique to achieve breakthroughs in the optimization of chemical processes.^325^,^334^ In this section, we present a concise overview of the most important recent applications of ML in catalysis with a special focus on the specific difficulties and challenges in the field.

#### 6.1.1. Machine learning approaches in chemistry

Machine learning is a rapidly growing field of computer science, where algorithms are trained to find empirical correlations in data. The concept of ML is conventionally attributed to the work by Arthur Samuel, dating back to 1959, who described an approach to teach machines to play checkers.^341^ However, one could track the seminal studies in machine learning to as early as the 1940s, when the first artificial neural networks (NN, see below) were introduced by McCulloch and Pitts.^342^ Although the first academic ML studies have appeared already more than 60 years ago, the peak in ML tools as a practical technology has been reached only in 2016 according to the research by Gartner Inc.^343^

ML approaches can be classified in a variety of ways based on the particular approach employed for solving practical problems. One of the widely employed classifications distinguishes supervised and unsupervised learning. For the latter, the learning is performed on a training dataset, in which only input values are provided to the algorithm without the corresponding output values. By contrast, the supervised learning is carried out on examples of input–output pairs with the possibility of generalization of the output prediction to an expanded or even different input dataset of a similar type. Another approach is to distinguish the ML strategies based on the type of predictions that the model delivers, which are classification, regression, clustering, dimensionality reduction and density estimation. In this case, we differentiate the type of task which a machine needs to accomplish, that is, to classify types of data, calculate some output from input values, separate data into different classes, reduce data descriptors or estimate distributions.^344^

**Fig. 13:**
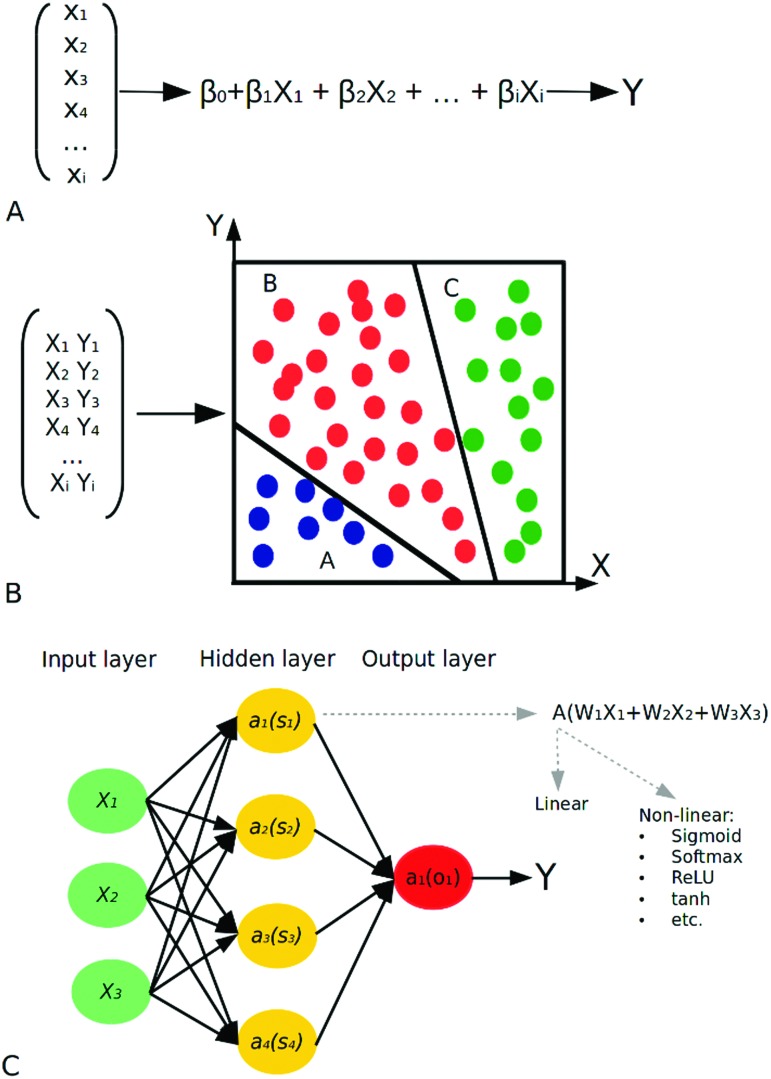
A schematic representation of the three main ML approaches often employed for modeling of catalytic processes: (A) linear regression, (B) support vector machine and (C) Neural Network approaches. In the linear regression (A) a linear relation is sought between a set of descriptors X_*i*_ and the activity measure Y. The support vector machine (B) processes the input descriptors X and Y and separates them into classes (A, B, C). The Neural networks (C) usually contain three types of layers: input, hidden and output. The model accepts the input parameters in the input layer, while the neurons of the hidden layers reevaluate these input parameters. Every neuron is a composition of an activation function *a*_*n*_ and a summatory *s*_*k*_. The output layer recalculates the results from the inputs and produces the final output of the network. The activation functions *a*_*n*_(*s*_*k*_) may be either linear or non-linear depending on the formulation of the network.

The basic ML approaches such as linear and logistic regression, support vector machine (SVM), decision trees, and random forest are well established in applied data science.^344^ The basic strategies underlying the most commonly employed methodologies are schematically illustrated in Fig. 13. In chemistry and catalysis, the selection of a particular ML strategy is commonly based on the type of task to be carried out. Linear regression (Fig. 13A) is a common strategy for QSAR/QSPR studies in drug design and QSPR studies in materials science and it is based on the idea that a specific descriptor – a specific measurable parameter – can be identified as an activity measure. The SVM model (Fig. 13B) operates in a multidimensional space that is built from various descriptors potentially reflecting the target characteristics. The separation of the input values in the multidimensional space can be achieved by linear, polynomial or hyperbolic functions (depending on which particular one is used as a kernel in the model).^345^ SVM models are used in data screening for the identification of efficient catalysts or porous materials with optimal adsorption characteristics.^345^–^347^ ML procedures are commonly applied to sufficiently large training datasets, making statistical procedures indispensable for estimating model performance and accuracy, and ultimately for determining the ML fitting properties. For example, principal component analysis (PCA) was employed to identify suitable descriptors for organometallic complexes.^348^,^349^

The most recent emergence of the Deep Learning concept resulted in widespread acclaim for ML technologies.^343^ Within this concept, artificial neural networks (NNs) are used to find patterns and correlations in the data (Fig. 13C). These networks are built from interconnected layers of neurons, which may formally resemble linear regression functions if the so-called linear activation functions are employed for their construction. However, nowadays a commonly accepted standard in the field necessitates the use of non-linear activation functions in the neurons. The output values from the neurons serve as the input for the next hidden layer or the output layer generating the final output of the NN. The NN is trained basically by providing the input data and reevaluating weight coefficients, which are (re)determined by the backpropagation algorithm initiated from the last hidden layer of the NN.

**Fig. 14:**
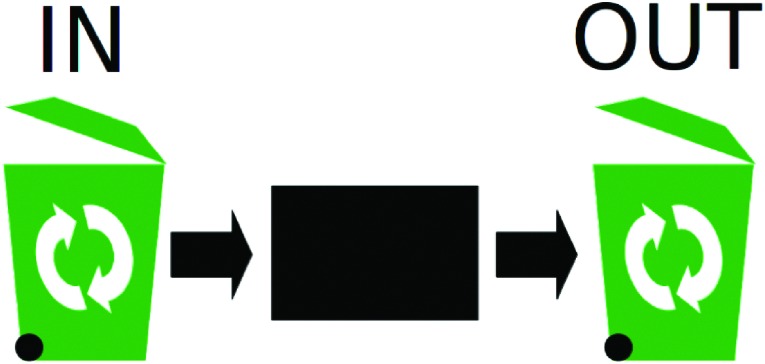
“Garbage In–Garbage out” problem in ML studies. If input values are inadequate, the model produces inadequate results.

Despite a variety of different ML approaches available nowadays, they all share some common challenges. The key one is actually shared with the more conventional modeling approaches. It is associated with the notion of “The Black Box” and can be illustrated by the “Garbage In–Garbage Out” problem (Fig. 14). Machine learning requires consistent input data for developing an adequate predictive model.^350^,^351^ Thus, the preparation of the datasets and the so-called feature engineering are the necessary steps in the construction of proper models for the ML studies. An intrinsic challenge for ML approaches is that the transparency in how the machine learns patterns or predicts properties depends on a chosen approach and cannot be fully achieved when, for example, neural networks are employed.^352^

#### 6.1.2. Descriptors for machine learning in chemistry

The identification of a digital parameter – descriptor – that reflects the target measurable property of a chemical system is the corner-stone of all ML approaches. Descriptor is a very general term and it can take a form of a digital representation of a molecule, material or any chemical system, its properties, structure (geometric or electronic) as well as that of any parameter of the environment. The examples of molecular or material descriptors are conventional chemical reactivity parameters such as the HOMO–LUMO gap, electron affinity and the d-band center, or a mathematical structural representation such as a connectivity matrix encoding chemical bonding in a molecule.^353^ Basically, any scalar or tensor that encodes in some manner the relevant property of a chemical compound, chemical system, or chemical environment may be used as a descriptor.

The mathematical representation of the geometric structure of an organic molecule ready to serve as an input for ML studies does not pose an issue nowadays. The presence of a carbon framework connected by strong covalent bonds in organic molecules makes it particularly attractive to employ the graph-based notations that can be backdated to Morgan's original idea of molecule representation in mathematical graphs.^354^–^356^ To date, several linear representations have gained a particular importance for the description of organic compounds. These are the so-called SMILES and InChI as well as the connectivity-based encoding formats implemented in MDL and XML files.^355^,^357^–^359^ These notations have become truly widespread, especially SMILES, with its various modifications in different areas of chemoinformatics.^360^,^361^

Although the utilization of these approaches can in principle be extended to inorganic and organometallic molecules, their direct representation faces a number of problems mostly related to the ambiguity of the bonding representation and consistent algorithms generally applicable to such chemical systems are substantially under-represented.^358^,^362^ Nevertheless, there are several ML studies where the successful utilization of the graph-based notation such as SMILES for the representation of simple organometallic molecules has been demonstrated.^357^,^363^–^365^ Catalytic systems based on inorganic and organometallic compounds are commonly characterized by the diversity of coordination polyhedra, stereoisomerism of the transition metal complexes, and the variability of the electronic nature of organometallic bonds. These are regarded as the key hurdles for the graph representation of the respective chemical systems and they have to be accounted for when applying the ML approaches to catalytic problems.

Previous sections clearly illustrate the key roles of electronic effects for the catalytic reactivity, rendering the associated parameters reflecting the electronic structure particularly important for the ML studies on catalytic systems. Besides the conventional chemical descriptors such as the atomic charges, Tolman's χ-factor, *etc*., several more comprehensive mathematical representations have been introduced so far. The simplest example of the related descriptors is the so-called Coulomb matrix that encodes the information about the electrostatic forces in a molecule. The diagonal elements of such matrices are a polynomial fit to the energy of free atoms constituting a given molecule. The off-diagonal elements correspond to the Coulomb repulsion energy values for all pairs of nuclei. This type of electronic structure representation was used, for example, for the ML prediction of the atomization energies of organic molecules.^327^,^330^ An alternative electronic structure descriptor was developed on the basis of the Fourier series of atomic radial distribution functions as an alternative to Coulomb matrices.^366^

The combination of steric and electronic descriptors is common in QSPR studies of organometallic compounds.^339^,^348^,^363^,^367^–^369^ Sigman and co-workers demonstrated that a combination of steric descriptors such as STERIMOL and Tolman cone angles together with the metal NBO charges can be used within ML approaches for predicting yields in homogeneous catalytic reactions.^369^,^370^ Density Functional Theory (DFT) methods may be effectively combined with the QSPR models to estimate the catalytic performance.^371^ The application of ML approaches to catalytic systems is commonly coupled with DFT calculations, as the DFT-computed molecular properties provide additional useful descriptors that can directly be employed in the training datasets.^305^,^329^,^335^

New computational approaches for the correct representation of transition metal complexes have been introduced recently. A notable example is the molSimplify open-source code developed by the group of Kulik.^372^ This computational toolkit has been designed to facilitate the generation of relevant structures and calculate properties of transition metal complexes. The program is based on the “divide and conquer” strategy, in which the organic ligand and the metal center are described separately. The implementation of an artificial NN in molSimplify allowed for the prediction of geometrical structures without the need for expensive geometry optimizations with conventional methods (such as DFT), while the combination of steric and electronic descriptors implemented in the code allowed for the prediction of electronic structure-related properties.^373^ This computational tool may be efficiently employed to aid in the design of new inorganic materials and transition metal-based catalysts.^362^,^373^

**Fig. 15:**
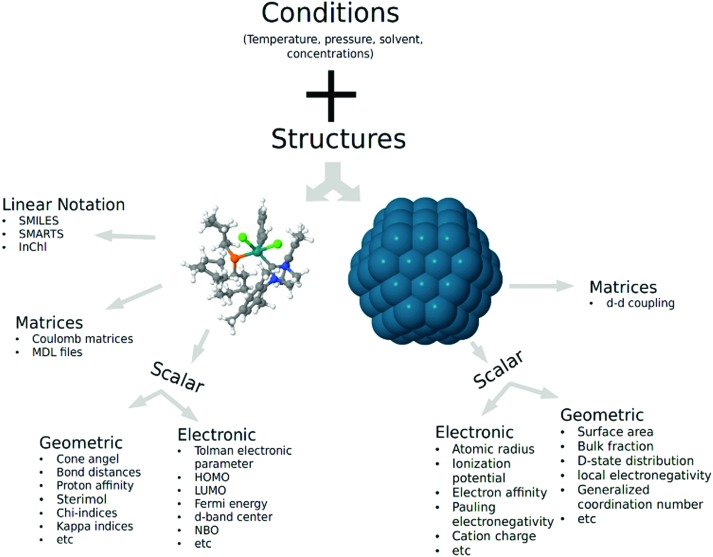
Chemical reactions can be represented by combinations of electronic and geometric structure descriptors with descriptors that encode the reaction conditions. One commonly distinguishes the descriptors designed for organometallic or organic molecular species and for bulk or nanoparticulate materials.

Chemical reactions and, even more so, the catalytic reaction mechanisms are intrinsically much more complex in representations compared to individual compounds when their representation as computer-processed data is considered. Most studies on chemical transformations employ combinations of descriptors corresponding to reactants, reaction conditions, catalysts, and efficiency metrics as conversion and yield (Fig. 15).^374^–^379^ There are special notations and file formats designed for the representation of organic chemical reactions such as the so-called SMARTS, which is the straightforward extension of the SMILES approach.^380^–^382^ Another useful method for the representation of chemical reactions is by encoding in an extended chemical data format such as an MDL RXN file.^383^ Besides this, it is worth mentioning the methods based on matrix transformations, such as the Dugundji-Ugi model, which is a formalism for representing chemical reactions based on BE- (Bond and Electron matrix) and R-matrices. The diagonal elements of the BE-matrix represent free valence electrons and off-diagonal ones correspond to bond orders between atoms. The R-matrix represents electron redistributions in the reactions; particularly, positive element values indicate bond formation and negative values indicate bond cleavage.^383^,^384^ Nevertheless, all of these digital formats are limited to organic reactions^385^ and they need to be substantially adjusted for catalytic applications commonly involving organometallic and inorganic components.

The comprehensive sets of descriptors that provide a sufficient representation of the specific chemical system are aligned with the measurable target characteristics, forming the datasets used in ML approaches. In practical applications, some crucial parameters of the chemical reactions may be omitted from the training datasets either because of the limited nature of the available information or for the sake of simplicity. It is important to realize that despite the growing volume of the available digitalized data on various catalytic systems reported in the scholarly literature, the construction of reliable datasets from the reported experimental data and proprietary databases is a general challenge.^385^ The mechanistic aspects of catalytic reactions, such as information about the transition states, intermediates and the respective energetics of elementary reaction steps, which were the focal point in the previous sections of this review, are rarely considered in ML. There are only a few studies accounting for transition states upon constructing the ML approaches for the prediction of chemical reactions.^322^,^383^,^385^ In experimental catalysis, catalytic tests commonly employ catalyst precursors, whereas the nature of the actual catalytic species is often unknown. Furthermore, the nature of the catalytic species may strongly depend on the activation procedure employed and/or evolve in the course of the catalytic reaction. Similar to computational modeling of catalytic reactions, the construction of adequate ML models and the selection of representative sets of descriptors still require a substantial human mechanistic insight into the fine details of chemical reactions.^370^ This results in the apparently conflicting requirements of availability of extended versatile training datasets, and detailed understanding of the crucial mechanistic parameters and their influence on the reactivity of different catalyst classes. This conflict is one of the most important conceptual challenges in the field.

### 6.2. Machine learning in catalysis

Many attempts to integrate ML methods into heterogeneous catalysis have been made in the last 3 decades. The first NN-based catalytic studies date back to the mid-1990s.^374^,^380^ Earlier applications of related methodologies could be found among the heterogeneous catalysis literature from the late 80s, which coincides with the peak of popularity of so-called expert systems.^386^ The essential properties of heterogeneous catalysts are surface area, elemental composition, and surface morphology – making these easily accessible characteristics attractive candidates for the descriptors. Most studies reported so far construct the training datasets by parsing the scholarly literature or by using in-house laboratory data.^374^,^376^–^379^,^387^ In general, the major part of ML studies in heterogeneous catalysis target two main challenges: (i) the direct prediction of catalytic activity (at the molecular level) or (ii) modeling of the chemical reaction efficiency, that is, to estimate indirectly the activity by building a model that relates the set of descriptors to the reaction yield or the reaction rate. The assessment of the intrinsic catalytic activity at the molecular or nanoscale is normally carried out by computing the adsorption energies or investigating the elementary reaction steps on surfaces. Here, the ML method is commonly used in concert with DFT modeling used to provide molecular-level insight and the necessary descriptors for the datasets. In this case, the ML provides the necessary predictive mechanism that effectively enables the transition from the microscopic DFT modeling to DFT-guided catalyst design. The indirect modeling of the catalytic efficiency mostly involves training of the ML models on the experimental datasets that combine descriptors related to the reaction conditions, nature, composition and physico-chemical characteristics of the catalysts, and those of the reagents. These two conceptually different approaches will be considered in more detail below.

#### 6.2.1. Modeling of catalytic reaction efficiency

The “popularity” of particular ML approaches in catalytic studies has evolved over time. Aligned with the general development of the artificial intelligence approaches, the first catalytic applications of ML employed expert systems, which attempted to emulate the decision making processes by “reasoning” through the available set of knowledge, following standard rule-based systems. For example, the so-called INCAP expert system was employed to design a promoted SnO_2_-based catalyst for the oxidative dehydrogenation of ethylbenzene.^386^ A later decline of expert systems, followed by the reintroduction of NN, shifted the paradigm. Notably, the same catalytic process was studied with the NN-based methods several years later and the great potential of this methodology for predicting reaction selectivities for related catalyst compositions has been demonstrated.^379^ The particular effectiveness of NNs has been shown for the applications in combinatorial catalysis. A representative example has been reported by Corma and co-workers, who employed an NN methodology to optimize a transition metal catalyst for the oxidative dehydrogenation of ethane.^388^ In practical applications, the representative training datasets used contain sets of descriptors related to the catalyst structure and reaction conditions (temperature, reaction time, and concentrations of reagents and products). In principle, the NN approach can be successfully employed for the identification of the optimal reaction conditions for a given catalytic system. A representative example is the earlier work by Sasaki *et al.* on NO decomposition over Cu-containing ZSM-5 zeolites.^374^ A conceptually similar approach has been employed for the NN-driven optimization of the alkene epoxidation by polymer-supported Mo(vi) complexes.^375^ In this work, the particle size and pore diameters were included as the key catalyst structure descriptors.

An NN-based or any other ML approach allows for the construction of predictive models that are suitable for optimizing catalyst formulation and conditions (the parameters included in the descriptor set) for highly complex processes without direct insight into mechanisms or knowledge of the specific atomistic details of the catalyst or the catalytic process under certain conditions. These conditions, in accordance with the basic principles of chemical engineering, are that a chosen combination of the descriptors that account for the catalyst structure, reaction conditions, and reaction efficiency (for example the reaction yield) adequately captures the key factors crucial for the particular catalytic process. For example, an NN based model has been successfully employed for analyzing the photocatalytic activity of TiO_2_ in the oxidative degradation of 17 α-ethynylestradiol.^387^ The NN model in this case accounted for the reaction conditions (catalyst and substrate concentrations) as well as the environment conditions such as water matrix conductivity and impurity (*e.g.* organic carbon) concentrations. This study has produced quite an intriguing and counter-intuitive insight that the impurity concentration had a comparable significance to the substrate concentration in the final ML model. The water matrix conductivity was found to be even more significant.

During the 2000s the focus of the applied catalysis community was significantly shifted towards the SVM approach, which has been widely employed to achieve accurate predictions of catalytic reaction efficiency for many systems. For example, the SVM and NN approaches in combination with genetic algorithms were used to predict the yield and selectivity of benzene isopropylation over H-beta zeolite catalysts. The results corresponded well with experimental data.^389^ A different SVM setup was employed for studying olefin epoxidation over a titanium silicate mesoporous catalyst.^345^ Markedly, the related ML models constructed based on the SVM approach to predict the outcome of the hydrothermal synthesis of hybrid organic–inorganic materials (such as MOFs) have been recently shown to yield results substantially outperforming conventional human experience-based strategies.^325^

Modeling of the efficiency of catalytic heterogeneous reactions based on experimental datasets is a developed field. In some cases such studies may have straightforward practical implications as, for example, the ML study by Akcayol and Cinar on the efficiency of a heated catalytic converter.^390^ The phenomenological approach that does not require the detailed mechanistic and structural information regarding the nature of the catalytic centers substantially facilitates the construction of very large training datasets containing macroscopic descriptors commonly employed in chemical engineering. The ML catalysis models constructed in this manner capture only the essential and (mostly) macroscopic physics and allow for a fast answer to a specific question. A thorough account of the mechanistic details of catalytic reactions for extended datasets of sizes large enough for ML studies was barely possible until very recently. This is because the respective information and the associated sets of descriptors could not be obtained with experimental techniques, given the requirement for the extremely resource- and time-consuming experiments, and the lack of broadly available open databases. Neither could this information be gained *via* computational modeling, as quantum chemical analysis of catalytic processes for extended catalyst libraries was well beyond the computing power of CPUs. However, such an approach fundamentally limits the predictive power of the ML outside the pre-defined classes of the catalytic systems. One naturally misses many possible catalyst design insights when using such ML models in line with the missing detailed mechanistic and structural information in the training datasets.

The solution to this natural drawback of the indirect ML approaches has emerged *via* their integration with the atomistic DFT modeling. Indeed the widespread of fast and semi-quantitatively accurate DFT methods together with the enormous progress in computational hardware witnessed in the last decade created a basis for this qualitative shift in catalytic applications of ML. This powerful combination, discussed below in more detail, holds promise as a practical approach for theory-guided ad-hoc catalyst design.

#### 6.2.2. Boosting DFT modeling of heterogeneous catalysts with machine learning approaches

The direct DFT modeling of complete reaction networks for different reactions over varied catalysts in a single study remains well beyond the current capacity of computational and human resources.^305^,^336^,^391^ Indeed, even geometry optimization of realistic catalyst models consisting of hundreds of atoms (slabs, nanoparticles, or supported metal clusters) may take considerable time to compute. Moreover, the mechanistic analysis of the competing reaction channels still requires the manual construction of model systems as well as the starting configurations of the reaction intermediates, and an initial guess for the transition states and/or the actual reaction steps. This is tedious work, and also the efficiency and even outcome often depend on the experience and skills of the researcher. Conventionally, DFT studies in catalysis consider rather limited sets of model systems and states and rather focus on formulating a conceptual understanding of chemical reactivity than generating data for the subsequent processing. Another limiting factor for the integration of quantum chemical modeling with ML was related to the limited accuracy of practical computational methods, which are usually hard to estimate *a priori*.^392^–^394^

There is no doubt that the big data analytics has the potential to provide technologies to greatly boost catalyst design. There is a clear demand for developing strategies alternative to the conventional quantum chemical modeling for providing the mechanistic details in an inexpensive and human bias-free manner to be employed in ML-based catalyst design approaches. The workaround to direct quantum chemical modeling in catalysis may be to employ ML methodologies also for generating the necessary mechanistic and microscopic information by training an ML model on the DFT-computed results. For example, the DFT calculations of binding energies of N, O, and NO species to various sites in Au–Rh-nanoparticles, clusters, and surfaces allowed for the construction of a linear regression model of the nanoparticle activity in the NO decomposition reaction with the so-called local structural similarity kernel as a key descriptor. In other words, the model in this study was trained on the descriptors constructed under the assumption of similar activity of sites with a similar local structure. The resulting ML model predicted activity of the bimetallic nanoparticles with variable size and composition and allowed for kinetic modeling of the direct NO decomposition process.^329^,^395^

**Fig. 16:**
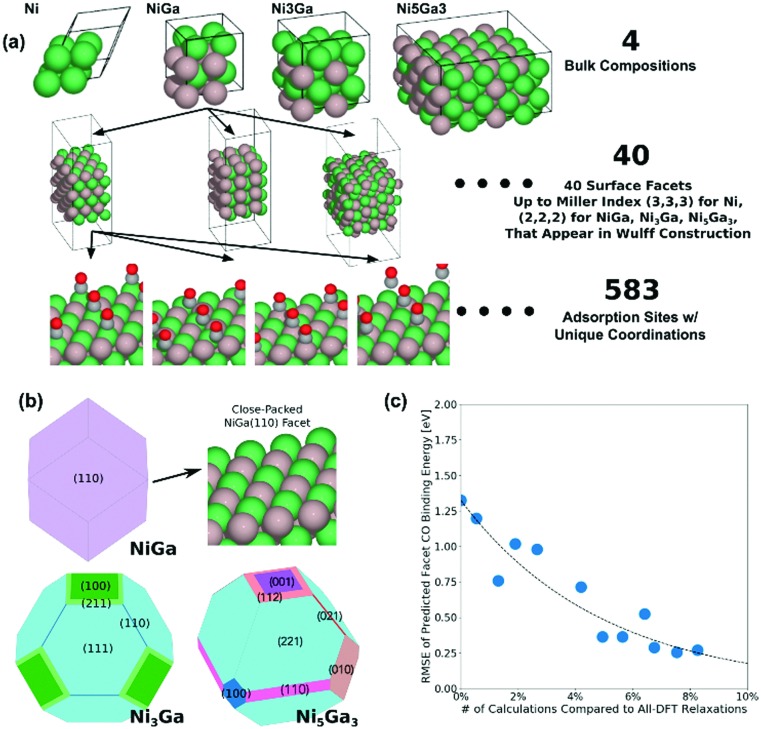
The application of ML approaches to predictive modeling of CO adsorption on bimetallic Ni–Ga nanoparticles. The associated model complexity stems from the large number of possible configurations emerging from (a) the wide variety of adsorption configurations and the configurations of the surface models along with (b) the variety of surface terminations available for the adsorption. By treating such a complex system using ML approaches, a substantial decrease (c) in the required CPU time of the adsorption energy predictions could be achieved compared to the conventional all-DFT geometry relaxation methods. Reprinted with permission from ref. 335. Copyright 2017 American Chemical Society.

Nørskov and co-workers developed an ML-based surrogate model that allows for a reduction in the number of necessary DFT calculations by an order of magnitude, while at the same time providing a means to model complex networks of surface reactions with sufficient accuracy. It has been applied, for example, to processes such as CO_2_ electroreduction on Ni–Ga-bimetallic nanoparticles and syngas conversion over a Rh(111) surface.^305^,^335^ Modeling of catalytic processes with bimetallic nanoparticles and exhaustive sampling of the attainable configurations may become extremely demanding in computational resources even if adsorption of a single simple intermediate such as the CO molecule is considered (Fig. 16a and b). The calculation of the adsorption energies of gaseous intermediates can be facilitated through the utilization of ML methods, which can reduce the number of necessary DFT computations to sample all relevant reaction pathways (Fig. 16c).

No significant accuracy deterioration should be expected when constructing ML models based on the DFT-computed energetics of heterogeneously catalyzed reactions. The Brønsted–Evans–Polanyi principle provides a practical means for estimating catalyst activity from the computed adsorption energies of key reaction intermediates. The study on the electrochemical reduction of CO_2_ over metal alloys employed an NN-based model that was trained on a dataset obtained from periodic DFT calculations. Such a model predicted the adsorption energies for reaction intermediates with an error of *ca.* 0.1 eV. Notably, by combining simple geometric and electronic structure descriptors such as local electronegativity, the effective coordination number of an adsorption site, ionic potential, electron affinity, and the Pauling electronegativity, a sufficiently high accuracy of the predicted energetics could be obtained.^396^,^397^

The use of a combined ML-DFT approach in computational catalysis achieves the accuracy of conventional DFT methods with a substantially lower computational demand, if a trained ML model is available. Recent studies demonstrate that well-trained ML models outperform hybrid DFT methods in the prediction of properties of organic molecules such as enthalpies and free energies of atomization, HOMO–LUMO energies, dipole moments, polarizabilities, zero point vibrational energies, heat capacities, *etc.*^323^ This suggests that it may become soon possible to develop an ML-only based computational procedure providing access to molecular-level information about chemical transformations that is cheaper and at the same time more accurate than the conventional quantum chemical methods. The key prerequisite for this is the availability of reliable experimental datasets to ensure the exhaustive training of such an ML model.

Datasets in heterogeneous catalysis are conventionally built from continuous operation that allows varying a limited number of parameters and obtain coherent data making it relatively straightforward to obtain large datasets from kinetic experiments. One can therefore anticipate the upcoming breakthroughs in heterogeneous catalyst design driven by the recent methodological progress in ML. Homogeneous catalysis studies conventionally deal with small scale batch experiments, where an individual reaction entry is a separate experiment that is not directly related to the other entries in a target dataset making it particularly challenging to generate larger datasets from the kinetic studies. The implementation of flow chemistry approaches to homogeneous catalysis studies may be viewed as one of the crucial ingredients towards the successful implementation of the big data strategy in this field. More important in our opinion is the development of broadly available open-access databases containing well-structured machine-readable catalytic activity datasets (that is every entry contains data necessary for training of ML models).

### 6.3. Open datasets as the basis for the catalyst design with machine learning techniques

Despite the many successful examples of the use of ML for addressing different scientific and technological problems reported in the past few years, there are several general problems that substantially limit the power and general applicability of ML-based approaches. The most important and the most generic problem that is particularly relevant for catalysis is the absence of comprehensive, large and publically-available datasets for training of efficient ML models. Despite the emergence of Big Data and the availability of large databases containing millions of chemical reactions, the majority of chemistry domains of catalysis still lack open datasets of appropriate size.^319^,^398^

**Fig. 17:**
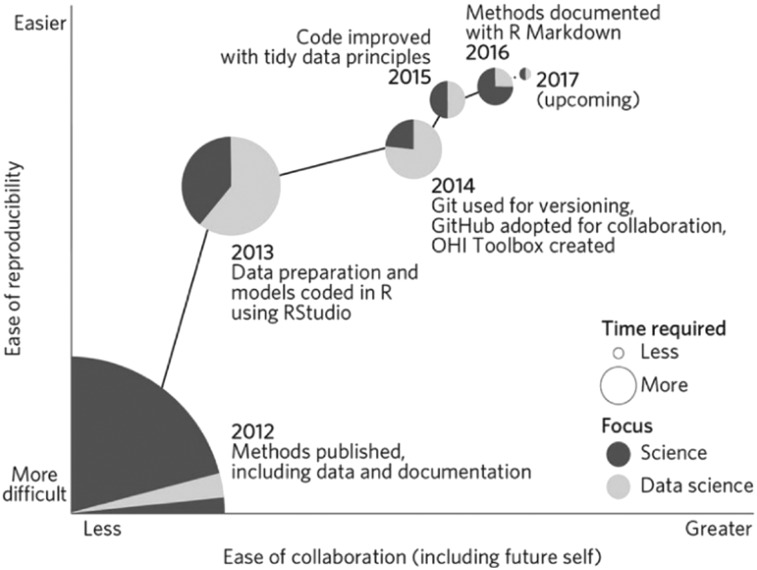
The influence of the open source software on the quality of the research results and its accelerating effect on the overall research progress within the Ocean Health Index (OHI) research, reprinted with permission from Macmillan Publishers Limited, 2017. Adapted from ref. 397.

There are several problems related to the proprietary nature of databases, difficulties with obtaining data from scientific articles and often-encountered cherry-picking (mis)practices.^385^,^399^ Addressing these problems is a general scientific challenge that spans well beyond the current subject of the development of ML approaches for catalysis design.^400^ Fortunately, practical measures are becoming available with the implementation of state-of-the-art software and methods for database organization. For instance, data nowadays can be organized with NoSQL technologies,^401^,^402^ which provide a straightforward method for the construction of databases that will include chemical reaction data on chemical environment parameters, reaction conditions, reactants, and products in a digital form. Combining the Open Access policy and user-friendly application programming interface to a database would enable sharing scientific data in a semi-automatic, fast and simple manner. Data in scholarly publications can be organized in a way facilitating the training of the ML models, by providing it in machine-ready table or CSV formats in the supplementary information. Such datasets should contain structural information on the employed catalysts and chemical compounds uniformly described through encoding in MDL files or using linear notations like SMILES and InChi.^403^ Furthermore, organizing the data according to the R Markdown format would make it ready for an algorithmic data analysis.^397^,^404^–^406^ Such a format facilitates reproduction of the results and verification of the correctness of statistical analysis.^407^Fig. 17 illustrates how the advanced information technologies such as Git workflow and R Markdown boosted the Ocean Health Index monitoring.^397^ We believe that algorithmic analysis-ready data science approaches could become transformative factors in catalysis design.

The scientific community steadily progresses towards the widespread implementation of the open access publishing policies driven by the clear socio-economic benefits and strong ethical implications.^408^,^409^ Publicly available data and instruments are transparent and can easily be controlled by the scientific community. The under-representation of negative scientific results in the scholarly literature is a well-recognized general problem^410^,^411^ and it is well known in all fields of catalysis sciences. The lack of negative data entries is an important problem for the development of practical ML instruments as they are the necessary component of a balanced training dataset and therefore are necessary to construct predictive models.^325^

### 6.4. Towards rational catalyst design with machine learning techniques

The pursuit of the general theory of catalysis that will give researchers a deep understanding of catalytic processes and a theoretical framework to anticipate new catalytic events has a nearly century-old history^412^ that has emerged in recent years in the general concept of the rational catalyst design. The rational design is a strategy for creating new structures with specific functions and properties based on a deep understanding of the fundamental factors that define the properties of interest. It is generally believed that the realization of this strategy in catalysis requires the understanding of how the catalyst structure and key chemical phenomena in catalytic processes are related to the observed activity.

While the progress in computational chemistry methodologies together with the availability of more and more powerful computational resources gradually enables modeling of any imaginable catalytic process, one still needs to construct a model system as well as to consider the relevant underlying chemical phenomena in the model. Therefore, any conventional computational modeling method by itself gives no deep understanding of the relation between the structure and underlying chemistry to the activity, and ultimately it is up to the skills of the researcher to select which predictions to make *via* the modeling. Machine learning-based modeling of catalytic reactions, in contrast, offers a way to enable a truly predictive modeling by virtue of its formalism.

As has been discussed above, a great number of descriptors are already available to model chemical processes involving transition metal catalysts. Theoretically, there is no fundamental limitation to compute these descriptors in an automatic way using already available cheminformatics software. By selecting a proper set of descriptors (either based on heuristic guess or *via* a trial-and-error approach), this would allow one to determine the key geometric and electronic properties of reactants and catalysts as well as the important environment parameters together with the optimal reaction conditions for a chemical process in question. The activity parameters such as TOF values, reaction yields, activation barriers for elementary steps could then be correlated with these descriptor-encoded key properties through the use of machine learning techniques. The accuracy of a trained ML model depends on the reliability and completeness of the data in the training dataset while the model generality depends on the size of the dataset.

The realization of the rational catalyst design strategy requires the descriptor representation that is detailed enough to be coupled with a training dataset that is sufficiently large and well-balanced. Indeed, the ideal case for rational design of, for example, electrocatalytic water splitting, the Fischer–Tropsch process, or CO_2_ hydrogenation catalysts would need to include the descriptor representation of all catalysts known to date and the corresponding activity data in the dataset. Moreover, the data on inactive catalysts have to be included explicitly to make the dataset balanced and the model predictive. This, in turn, would allow estimating the activity of a catalyst that is not yet synthesized or to determine descriptor values that maximize the activity. The latter would enable the targeted synthesis of catalysts having the desired properties. Proper accounting for the catalyst poisoning and degradation while training ML models will allow *a priori* tuning of the optimal reaction conditions.

Therefore, the availability of consistent open-access databases on catalyst activity is of paramount importance for the rational catalyst design with ML approaches. The promotion of open-access policies in scientific data publishing and active use of new tools for digital data representation enabling automated computer data analysis are thus the crucial ingredients towards the realization of such an ML-based rational design strategy. The proliferation of data science technologies to catalysis research is therefore much anticipated. Because of the recent successes in prediction of organic reactions,^326^,^385^,^413^,^414^ properties of materials,^321^,^331^,^336^ and the well-known utility of the QSAR/QSPR approach in drug design, there is a strong indication that the ML-based rational catalyst design is about to emerge.

## 7. Conclusions

Rational catalyst design – the concept by which a successful catalytic system could be forecasted based on the results of only computations – has long been and still remains the “Holy Grail” of heterogeneous catalysis. An opportunity to avoid or even to just minimize tedious and costly experimental search for the optimal composition of multicomponent catalysts and reaction conditions for a given chemical transformation by replacing it with some computer-based algorithm capable of directing this search is very attractive and if practically realized holds a promise of revolutionising chemical science and technology. Despite great progress witnessed during the last two decades in the development of new approaches for theory-guided catalyst development, substantial new methodological advances are still necessary to enable their widespread implementation in the daily lives of catalysis researchers.

One of the most important shortcomings of the established computational strategies is the dominance of the basic 0 K/UHV approximation commonly employed for the development of mechanistic concepts in catalysis. Despite being capable of providing a satisfactory mechanistic description of a catalytic phenomenon, they often lack sufficient predictive power mostly due to the inability to adequately account for the crucial physical effects encountered under the conditions of actual catalytic processes. The understanding of such phenomena and their impact on the molecular-level processes underlying the performance of catalytic systems is one of the key challenges to realization of the catalysis by design approach.

In this review, we have discussed some important recent methodological developments enabling the transition from the 0 K/UHV to *operando* computational modelling. The importance of this transition was highlighted by discussing how the molecular level picture of the catalytic sites and the associated reaction mechanisms evolve drastically when a correct account for chemical environment, pressure and temperature effects is given in the molecular simulations.

An important challenge in modern heterogeneous catalysis is to reveal the nature of the active sites and to understand how their structure evolves when exposed to the realistic catalytic environments and temperatures. In the first sections of this review, we discussed computational approaches allowing comprehensive sampling of the complex chemical space to determine the potential active site candidates and construct representative active site models as well as to assess their thermodynamic stabilities as a function of the reaction conditions. We discussed how global optimization (GO) techniques can be used to address the basic structural problem in heterogeneous catalysis. These methods can be efficiently used to screen candidate structures and automatically search for stable active site formulations. This was followed by the discussion of the constrained *ab initio* thermodynamic analysis (AITD) approaches for assessing the thermodynamic stabilities of different active site ensembles under the varying reaction conditions. Indeed, the integration of GO and AITD methods has proven useful in isolating relevant structures under realistic conditions for a number of systems, from gas phase particles to oxide surfaces, and the combination of the two techniques is becoming commonplace. Obtaining relevant structures of the active site is, however, just a prerequisite for a reliable description of the catalytic process, with reactant concentration, temperature, pressure or presence of solvent to be accounted for. This situation corresponds to studying catalytic processes on the free energy landscape, which is a generic problem of computational chemistry and was tackled in the next section by discussing the use of Hessian-based as well as advanced *ab initio* molecular dynamics (AIMD) approaches. These techniques are already quite routinely applied for studying mechanisms of catalytic reactions, either by open-ended searches of the free energy surface, for example with metadynamics, or by integration with GO methods through free energy path search techniques. It can be seen that the state of the art in both GO and AIMD are converging towards *operando* descriptions of complex reactions on multicomponent systems. In GO, the reactive global optimization (RGO) method allows for a kinetics-based description of the reaction network for supported catalysts along with adsorbates, under pre-defined conditions. In AIMD, complex, multidimensional collective variables allow for a broad sweep of the reaction network for catalysts of similar complexity to that of RGO, and the discovery of new mechanisms. In both cases, the limitations are the computational expense of the calculation methods. One possible route to alleviate this problem is, in our opinion, the development of machine learning-based potentials, which are more robust, transferable and can adequately handle chemical transformations.^391^,^415^,^416^

These endeavours inevitably lead to the generation of large volumes of mechanistic data and insights, which can become so complex and heavy that it is no longer possible to rely solely on the human ability to analyse and rationalise them. New approaches that would limit the human bias in analysis and at the same time provide with the means to extract the experimentally verifiable parameters from the microscopic data are becoming crucial. In this context, the conventional chemical engineering reductionist approach in the form of microkinetic modelling or kinetic Monte Carlo becomes instrumental to reduce the mechanistic complexity to a tangible number of experimentally verifiable parameters suitable for guiding the experimental catalyst development and process optimization efforts. Kinetic modelling is naturally well integrated with any method which provides energetic data about minima and transitions states, such as GO, AITD or AIMD. One current challenge for the increased adoption of engineering approaches by computational chemists is to increase the sophistication of the models. Moving beyond mean-field descriptions to models with proper adsorbate interactions, coverage dependences and substrates which change under the conditions of the reaction is important to match the complexity of *operando* catalysis. An alternative approach that has gained importance and attention recently in basically all areas of human activities including chemistry and catalysis relies on a machine to not only generate the numerical data but also analyse and guide the research and development efforts. Ideally, machine learning would allow removing completely the human bias from the model formulation, data analysis and expanding the scale at which the analysis is carried out to drive innovation in catalysis research. However, these idealistic views are still very far from coming true. The success of machine learning approaches in catalysis sciences still heavily relies on the definition of suitable descriptors and on the quality of the available datasets. Human interference is still often a necessity for pre-processing the data to assess its quality and to adjust it for subsequent construction of the ML algorithms. Having said this, we are confident that ML approaches for the data analysis will gain importance in computational catalysis research and will find many applications not only in identification of trends enabling the search for improved catalysts outside the conventional scopes, but also as the means to facilitate the very basics of the computational catalysis that is electronic structure calculations and statistical thermodynamic analysis.

The main features of individual methods discussed in this review are summarized in Table 1. It has been already mentioned that subsequent or even simultaneous application of all methods summarized in Fig. 1 would be computationally prohibitive. However, for many catalytic systems it is not critical to apply all extensions: for example, (i) catalysts with known structure do not require GO methods, (ii) catalysts with an inert structure and low concentration of active sites do not require AITD methods, (iii) catalysis at a gas phase interface with low reactant concentrations could be treated without biased MD methods. For individual extensions beyond the reference 0 K/UHV model, Table 1 shows under which conditions each method should be applied, gives a few examples and some comments with respect to their practical use. It is our hope that this review helps readers to better understand the principles and applicability of methods that extend beyond the 0 K/UHV model towards computational *operando*.

**Table 1:** The computational approaches discussed in the review, with appropriate types of problems, examples of relevant system classes and general remarks on key issues and limitations

	Application guidelines	Example of catalytic system classes	Remarks
Global optimization	Unknown catalyst structure (known composition, low *T*, low *P*).	Subnanometre metallic clusters on inert substrates/metal oxide surfaces for low *T* oxidation catalysis.	Number of structures scale exponentially with system size.
			MC methods preferred for maintaining local structural information during search (pathways).
	Unknown catalyst structure (unknown composition, any *T* or *P*).	Dynamically restructuring reducible oxide surface catalysts in an O_2_ atmosphere.	Couple with AITD for unknown system stoichiometry.
*Ab initio* thermodynamics	Unknown structure of catalyst surface.	Phase diagram of metal oxide catalyst surfaces.	Only modest computational requirements when vibrational contributions to surface free energies are neglected.
	Morphology/growth direction (using Wulff construction).	Nanoparticle shape (nanoalloys/metal carbides).	
Biased molecular dynamics	Reactions on liquid/solid interface.	Electrochemical reduction on metal surfaces.	Typically on the order of 10^5^ MD steps (force evaluations) per elementary reaction.
	High reactant concentration.	Heavy oil hydrogenation on metal carbide nanoparticles.	Choice of proper collective variable is an issue.
	High reaction temperature.	NOx reduction over metal-exchanged zeolites.	Two MD approaches to choose from: Born–Oppenheimer or Car–Parrinello.
	Competing species in reactive mixture.	Methanol-to-olefin process in nanoporous solid acids.	Kinetic information is accessible.
	Loosely-bound complexes of reactants/TS/products with catalyst.	Hydrocarbon cracking in acid zeolites.	Only fixed composition runs in the *NVT* ensemble done so far.
Microkinetic modelling	Complex reaction networks.	Partial (de)hydrogenation reactions of unsaturated hydrocarbons on metal surfaces.	Lateral interactions are rarely accounted for due to the mean-field approximation.
	Translation of molecular-level mechanistic data into directly measurable macroscopic kinetic parameters.	Alkylation over FAU zeolites/NH_3_ synthesis over metal catalysts.	Algorithms to include secondary processes (*e.g.* catalyst surface reconstruction, long-term deactivation, *etc.*) are not available.
Machine learning	Availability of experimental dataset on catalytic activity.	NO decomposition over Cu-containing zeolites.	Straightforward account for reaction conditions is possible.
	Easy construction of the training dataset *via* DFT computations and the system too complex for DFT-only modeling.	Binary metal alloy catalysts (*e.g.*, Au–Rh or Ni–Ga).	Adequacy of the training data determined by the accuracy of the DFT computations (garbage in-garbage out principle).
			Lack of open-access comprehensive databases on catalytic activity.
			Lack of data related to negative catalytic results.

## Abbreviations

UHV: Ultrahigh vacuum
PES: Potential energy surface
MD: Molecular dynamics
GO: Global optimization
BH: Basin hopping
MH: Minima hopping
EA: Evolutionary algorithm
NEB: Nudged elastic band
DFT: Density functional theory
GM: Global minimum
MP2: Second order Moller–Plesset perturbation theory
HAGA: Hybrid *ab initio* genetic algorithm
STM: Scanning tunneling microscopy
KMC: Kinetic Monte Carlo
RGO: Reactive global optimization
AITD: *Ab initio* thermodynamics
LDA: Local density approximation
PBE: Perdew–Burke–Ernzerhof (exchange correlation functional)
SCR: Selective catalytic reduction
AIMD: *Ab initio* molecular dynamics
MTD: Metadynamics
ITS: Integrated tempering sampling
CPMD: Car–Parinello molecular dynamics
TI: Thermodynamic Integration
QCT: Quasiclassical trajectory
TPS: Transition path sampling
ZPVE: Zero-point vibrational energy
MTO: Metal-to-olefin
HTST: Harmonic transition state theory
MKM: Microkinetic modelling
TST: Transition state theory
DRC: Degree of rate control
MLM: Machine learning
NN: Neural networks
SVM: Support vector machine
QSAR: Quantitative structure–activity relationships
QSPR: Quantitative structure–property relationships
CPU: Central processing unit
GPU: Graphics processing unit
PCA: Principal component analysis
SMILES: Simplified molecular-input line-entry system (molecular representation format)
InChI: International Chemical Identifier (molecular representation format)
MDL: MDL Information Systems, Inc.
XML: Extensible markup language

## Conflicts of interest

There are no conflicts to declare.
